# Mechanistic insights into ENO2-driven glycolysis and tumor progression in hepatocellular carcinoma

**DOI:** 10.1371/journal.pone.0361057

**Published:** 2026-10-27

**Authors:** Yan Liu, Xiao-Ran Zhang, Li Dai, Hai-Liang Li, Xiang-Ren Jin, Hong-Bin Qi, Ke Tang, Qiang Li

**Affiliations:** 1 Guizhou Provincial People’s Hospital, Department of Hepatobiliary Surgery, Guiyang, Guizhou Province, China; 2 Department of Surgery, The Affiliated Hospital of Guizhou Medical University, Guiyang, Guizhou Province, China; 3 Guizhou Medical University, School of Basic Medical Sciences, Anshun, Guizhou Province, China; 4 Guiyang Nanming District People’s Hospital, Department of Surgery, Guizhou Province, China; 5 Department of Surgery, The First People’s Hospital of Zunyi, The Third Affiliated Hospital of Zunyi Medical University, Zunyi, Guizhou Province, China; Wayne State University, UNITED STATES OF AMERICA

## Abstract

**Background and Aims:**

Neuron-specific enolase 2 (ENO2) has been implicated in tumor metabolism, but its biological role in hepatocellular carcinoma remains incompletely defined. This study aimed to investigate the expression, prognostic relevance, and functional role of ENO2 in HCC.

**Methods:**

Public transcriptomic datasets, RNA sequencing, WGCNA, survival analyses, and pathway enrichment analyses were integrated to identify HCC-associated genes. ENO2 expression was validated in HCC cell lines and paired clinical tissues.Gain- and loss-of-function, catalytic-rescue, glycolytic-inhibition, and pyruvate-rescue experiments were performed to evaluate glycolytic activity, proliferation, colony formation, migration, invasion, and tumor growth.

**Results:**

ENO2 was upregulated in HCC and was associated with advanced tumor stage and unfavorable survival outcomes in public datasets. ENO2 overexpression increased glycolytic activity and malignant phenotypes, whereas ENO2 depletion produced opposite effects. In ENO2-depleted cells, re-expression of shRNA-resistant ENO2 wild type restored the impaired glycolytic and malignant phenotypes, whereas the catalytic-deficient ENO2 mutant failed to restore them. Moreover, 2-deoxy-D-glucose attenuated ENO2 overexpression-associated phenotypes, while pyruvate partially rescued phenotypes associated with ENO2 depletion. ENO2 expression was also associated with altered PI3K and AKT phosphorylation.

**Conclusions:**

ENO2 may contribute to HCC progression by enhancing glycolytic metabolism and malignant phenotypes, partly in association with PI3K/AKT signaling. ENO2 is a candidate metabolic and prognostic biomarker that requires validation in larger independent clinical cohorts and warrants further investigation as a therapeutic candidate.

## 1 Introduction

Hepatocellular carcinoma (HCC) develops through a complex interplay of multiple genes and signaling pathways, with metabolic reprogramming as a central characteristic [1,2].Cancer cells in the tumor microenvironment adjust to hypoxia and nutrient scarcity by altering glucose, lipid, and amino acid metabolism to satisfy the energy and biosynthetic needs of their rapid proliferation [3,4].This metabolic shift is meticulously controlled by crucial signaling pathways such as PI3K/AKT/mTOR, Ras-Raf-MEK-ERK-cMYC, and LKB1-AMPK, creating a dynamically integrated metabolic-signaling network.ENO2 is a glycolytic enzyme that catalyzes the conversion of 2-phosphoglycerate to phosphoenolpyruvate. Previous studies have implicated ENO2 in HCC through several regulatory mechanisms. For example, ENO2 expression has been linked to hypoxia-associated transcriptional programs, the lncRNA TUG1/miR-122-5p axis, and the circESYT2/miR-665 axis. More recently, an H4K5 lactylation–ENO2 positive-feedback loop has also been proposed in HCC [5]. These studies provide important evidence that ENO2 is involved in HCC metabolic reprogramming and tumor progression. However, they primarily focus on individual upstream regulatory mechanisms. A systematic strategy integrating co-expression-network analysis, exploratory transcriptomic screening, and glycolysis-related gene sets to prioritize ENO2, together with functional evaluation of its catalytic contribution and downstream pathway-associated phenotypes, remains insufficiently characterized.The PI3K/AKT pathway, often activated in hepatocellular carcinoma, is crucial for regulating cell growth, metabolism, survival, and motility [6–8].Dysregulation of this pathway profoundly reshapes the metabolic landscape of HCC cells by affecting downstream effectors such as mTOR, HIF-1α, and c-Myc, including enhancing glycolysis, promoting de novo lipid synthesis, and altering amino acid metabolism [9–11].Previous evidence suggests potential crosstalk between ENO2-associated metabolic regulation and PI3K/AKT pathway activity. Activation of the PI3K/AKT pathway can enhance ENO2 activity. In pancreatic ductal adenocarcinoma, the PI3K/AKT/mTOR pathway activation by insulin-like growth factor-1 leads to histone deacetylase 3-mediated deacetylation at the ENO2 K394 site, which enhances ENO2 enzymatic activity and glycolysis, promoting cancer metastasis [12]. Conversely, ENO2 may indirectly influence PI3K/AKT pathway activity through metabolic remodeling and tumor microenvironmental adaptation. In hepatocellular carcinoma, BNIP3-mediated mitophagy enhances glycolytic flux via the AMPK–ENO2 signaling axis, thereby providing lenvatinib-resistant cells with a competitive survival advantage [13]. In addition, AMPK and PI3K/AKT signaling are functionally interconnected in the regulation of tumor metabolism, cell survival, and therapeutic resistance [14]. Tumor microenvironment acidification has been shown to activate PI3K/AKT signaling, upregulate stearoyl-CoA desaturase 1, and promote lipid accumulation in HCC cells [15]. Nevertheless, the functional requirement of PI3K/AKT signaling for ENO2-associated malignant phenotypes in HCC requires further experimental clarification.Recent bioinformatics-driven studies have increasingly identified prognostic and functionally relevant genes in HCC through integrated computational analyses followed by experimental validation. For example, Wei et al. identified SF3A3 as a prognostic factor through integrated single-cell landscape analysis and showed that SF3A3 knockdown suppressed HCC cell proliferation and migration, partly through PI3K/AKT signaling [16]. In addition, EXOSC10 and DNAJC1 have been characterized as potential prognostic biomarkers associated with HCC cell proliferation and migration [17,18]. CSNK1E was reported to regulate HCC cell proliferation and migration [19], whereas EXOSC3 knockdown induced G1/S arrest and inhibited HCC cell proliferation [20]. Collectively, these studies support the value of integrating bioinformatics-based candidate prioritization with experimental functional validation in HCC. Building on this research framework, the present study specifically focused on ENO2 as a glycolysis-related candidate and evaluated its associations with glycolytic metabolism, malignant phenotypes, and PI3K/AKT pathway-associated signaling.In the present study, we integrated weighted gene co-expression network analysis (WGCNA), exploratory RNA sequencing of three paired HCC and adjacent non-tumor tissues, and glycolysis-related gene-set analysis to prioritize ENO2 as a candidate regulator of HCC progression. We subsequently evaluated the clinical relevance of ENO2 and investigated its effects on glycolysis, proliferation, migration, invasion, and tumor growth using in vitro and in vivo models. In addition, we examined whether pharmacological inhibition of PI3K/AKT signaling using LY294002 modulates ENO2-associated phenotypes. Thus, this study extends previous ENO2 research in HCC in three respects: (i) it systematically identified ENO2 through the intersection of WGCNA, RNA-seq, and glycolysis-related gene-set analyses; (ii) it provided the first functional validation of ENO2 in an orthotopic liver tumor model of HCC; and (iii) it used LY294002 experiments to examine whether PI3K/AKT pathway activity contributes to ENO2-associated phenotypes.These findings provide additional evidence supporting ENO2 as a candidate metabolic biomarker and therapeutic target in HCC.

## 2 Materials and methods

### 2.1 Weighted Gene Co-expression Network Analysis (WGCNA)

The datasets GSE45267, GSE101685, and GSE84402 were combined into a single expression matrix using the “inSilicoMerging” package in R (version 4.2.1) after eliminating batch effects. Subsequently, a co-expression network was constructed, and hierarchical clustering (minimum module gene number = 30) was used to generate a clustering tree. After setting the soft threshold β = 5, the distance between module eigengenes was calculated, ultimately yielding 29 stable co-expression modules. Key genes were screened by comparing the correlation between gene expression in each module and the “Tumor” eigenvector. Based on the criterion |MM| > 0.8, 143 key genes were identified in the “Brown” module.

### 2.2 Pan-cancer analysis

Pan-cancer RNA-seq data in TPM format and associated clinical information were sourced from the TCGA-ALL project. Visualization was performed using ggplot2, while univariate Cox regression and the forestplot package in R (version 4.2.1) were utilized to create forest plots evaluating ENO2’s prognostic impact on various cancers.

### 2.3 Analysis of gene expression and tumor clinical characteristics

Transcriptomic data from 371 HCC tissues and 50 adjacent tissues in TCGA were integrated with 110 normal liver samples from GTEx. Differential expression analysis was performed using the limma and stats packages, with visualization by ggplot2. Survival analyses were conducted using the survival and survminer packages. Pathway activity was estimated by single-sample gene set enrichment analysis, and correlations between ENO2 expression and pathway scores were evaluated. ENO2 protein expression was examined using the Human Protein Atlas database.

### 2.4 RNA-seq analysis

Patient tissue samples were accessed for research purposes on 16/04/2025. RNA was extracted from patient tissue samples, and its concentration, purity, and integrity (RIN value) were detected. Raw sequencing reads were assessed using FastQC. Adapter sequences and low-quality reads were removed using fastp. Clean reads were aligned to the human reference genome using HISAT2, and gene-level read counts were generated using featureCounts. Differential expression analysis was performed using DESeq2. Genes with |log2 fold change| > 1 and adjusted P value < 0.05 were considered differentially expressed.Following quality control, libraries were constructed and sequenced using the Illumina platform. The “DESeq2” package in R was used to compare gene expression differences between groups and screen for differential molecules. Following ID conversion, the clusterProfiler package in R 4.2.1 was utilized for tumor immune pathway enrichment analysis, and the findings were visualized using ggplot2.

### 2.5 Collection of human tissue specimens

Eight paired HCC and adjacent non-tumor tissues were collected from patients at the a tertiary medical center. None had received chemotherapy, radiotherapy, biological therapy, or traditional Chinese medicine before surgery. Human tissue specimens were accessed for research purposes on 27/04/2025. Written informed consent and institutional ethics approval were obtained. Samples were used for qRT-PCR, Western blotting, and immunohistochemistry. Because complete clinicopathological information was unavailable for all archived specimens, they were not used for clinicopathological or survival analyses.

### 2.6 Cell culture and transfection

HepG2, Huh7, SNU387, SNU398, and THLE-2 cells were obtained from the American Type Culture Collection (ATCC), and LM3 cells were obtained from the Liver Cancer Institute of Fudan University. THLE-2, an authenticated normal hepatocyte line, was used as the liver-derived reference cell line. SNU398 and SNU387 cells were cultured in RPMI-1640 supplemented with 10% fetal bovine serum and 1% antibiotics, The remaining cell lines were cultured in DMEM supplemented with 10% fetal bovine serum and 1% antibiotics. All cells were maintained at 37°C in a humidified atmosphere containing 5% CO_2_ and were routinely tested for mycoplasma contamination.

The siRNA sequences were as follows: ENO2 siRNA NC, 5′-CACTGAAGGTGGAGGTCTTCACATC-3′; ENO2 siRNA 1#, 5′-GCGTTACTTAGGCAAAGGTGTGGCA-3′; ENO2 siRNA 2#, 5′-CCCACAGTGGAGGTGGATCTCTATA-3′; and ENO2 siRNA 3#, 5′-GGTGGATCTCTATACTGCCAAAGGT-3′. Cells were transfected with siRNAs using Lipofectamine 3000. Lentiviral vectors for negative control, ENO2 overexpression, and ENO2 shRNA were generated by GeneChem. Transfection and infection were performed according to the manufacturers’ protocols.

### 2.7 qRT-PCR

RNA was isolated from HCC cell lines and tissues using TRIzol reagent (Invitrogen, USA), converted to cDNA, and utilized in further experiments. DNA amplification utilized TB Green® Premix Ex TaqTM (Takara, Japan) and was detected on a CFX96TM real-time quantitative PCR system (Bio Rad, USA).

The primers used in this study are:

ENO2 forward primer: 5′ AGCCTCTACGGGCATCTATGA 3′
Reverse primer: 3′-TTCTCAGTCCCATCCAACTCC – 5′
The forward primer for GAPDH is 5′-CCACAGTCCATGCCATCACTG-3′.
Reverse primer: 5′ GTCAGGTCCACCACTGACACG 3′.

GAPDH served as the internal reference. The 2 − ΔΔCt method was used to calculate the results.

### 2.8 Western blot

Proteins were extracted using RIPA buffer, quantified with a BCA kit, separated by electrophoresis, and transferred to PVDF membranes. Membranes were blocked with 5% skim milk and incubated with primary antibodies overnight at 4°C, followed by HRP-conjugated secondary antibodies. Bands were visualized using a Bio-Rad imaging system and quantified with ImageJ. ENO2, PI3K, AKT, and GAPDH were normalized to GAPDH, whereas p-PI3K and p-AKT were normalized to total PI3K and AKT, respectively.

Antibodies included: anti-ENO2 (1:1000, Proteintech, #10149–1-AP),
anti-Pan-AKT (1:2000, ABclonal, # A18675),
anti-PI3 Kinase (1:1000, ABclonal, # A22487),
anti-Phospho-AKT (1:2000, ABclonal, #AP1430),
anti-Phospho-PI3K (1:1000, ABclonal, #AP0427),
anti-GAPDH (1:5000, Proteintech, # 60004–1-Ig),
HRP goat anti-rabbit IgG (1:2000, Proteintech, #SA00001–2),

### 2.9 Cell viability assay

LM3 and SNU398 cells were seeded in 96-well plates at 5,000 cells/well. Cell viability was measured using a CCK-8 kit at indicated time points, and absorbance was recorded at 450 nm.

### 2.10 EdU probe labeling proliferation assay

Cells were seeded in 12-well plates at 20,000 cells/well and incubated with 20 μM EdU for 2 h. After fixation, cells were stained with DAPI and iF555 according to the manufacturer’s instructions. EdU-positive cells were counted under a fluorescence microscope.

### 2.11 Clone formation assay

The number of treated HCC cells was adjusted to 1 × 10³ cells/well, gently resuspended in medium, and added to a 6-well plate, seeded once per well, and placed in an incubator for about 2 weeks. Cells were fixed using 4% paraformaldehyde, stained with 0.25% crystal violet, and then the clones were counted and photographed.

### 2.12 Wound healing assay

LM3 or SNU398 cells were seeded in a 6-well plate at an appropriate density. Upon reaching 90–100% confluence, a scratch was uniformly created using a 200 μl pipette tip. Cell migration ability was assessed by measuring changes in scratch area at three time intervals: 0, 24, and 48hours.

### 2.13 Migration and invasion assay (transwell)

Transwell chambers (CoStar, USA) were prepared. The upper chamber received 200 μl of serum-free medium with 1 × 10^4^ cells, optionally mixed with Matrigel matrix, while the lower chamber was filled with 800 μl of medium containing 10% FBS. Cells were incubated in a 37°C, 5% CO_2_ incubator for 24–36 h, washed twice with PBS, then fixed and stained.Cells that migrated or invaded were quantified using microscopy.

### 2.14 Immunohistochemistry (IHC)

Paraffin-embedded tissue sections were deparaffinized, rehydrated, subjected to antigen retrieval, and blocked for endogenous peroxidase activity. Sections were incubated with ENO2 primary antibody overnight at 4°C, followed by secondary antibody incubation, DAB development, counterstaining, and microscopic evaluation.

### 2.15 Extracellular Acidification Rate (ECAR)

Extracellular acidification rate (ECAR) was measured using a Seahorse XF96 analyzer. HCC cells were seeded in Seahorse XF96 plates at 4 × 10⁴cells per well 24 h before analysis. Before measurement, cells were washed and incubated in assay medium containing 2 mmol/L glutamine at pH 7.4 in a CO_2_-free incubator. Glucose, oligomycin, and 2-deoxyglucose were sequentially injected to assess glycolytic activity, glycolytic capacity, and non-glycolytic acidification. Data were analyzed using Wave software.

### 2.16 Glucose uptake detection

Glucose consumption in culture medium was measured using a glucose oxidase colorimetric assay. Supernatants were centrifuged to remove debris, incubated with kit reagents, and measured at 505 nm. Glucose consumption was calculated from the standard curve.

### 2.17 Lactate level detection

Lactate levels in culture supernatants were quantified using a lactate dehydrogenase-coupled colorimetric assay. Samples, standards, and blanks were incubated with reaction buffer, enzyme, coenzyme, and chromogenic reagents according to the manufacturer’s instructions. Absorbance was measured at 450 nm.

### 2.18 ATP level detection

Intracellular ATP levels were measured using an ATP Assay Kit (solarbio, BC0300) based on the NADH rate method, according to the manufacturer’s instructions. Briefly, cells were harvested and lysed using the supplied lysis buffer. The lysates were subjected to the enzymatic reaction, and the change in absorbance at 340 nm was monitored using a microplate reader. ATP levels were calculated based on the rate of NADH formation and normalized to the total protein concentration of the corresponding samples. The results were expressed relative to the control group.

### 2.19 In vivo experiments

BALB/c nude mice, aged six to seven weeks, were obtained from Collective Pharmachem. The rearing environment was controlled at 22 ± 1°C with 50 ± 1% relative humidity and a 12/12 h light/dark cycle. For the subcutaneous xenograft model, mice were subcutaneously inoculated with 2 × 10⁶ cells in the right armpit and randomly assigned to 5 groups, each containing 5 mice. Tumor volume was consistently assessed using a vernier caliper and calculated with the formula: tumor volume = length × width^2^ / 2.The experiment lasted for 7 weeks.

Humane endpoints were predefined before the start of the experiment, and mice were euthanized if the maximum tumor diameter approached or exceeded 15 mm, corresponding to an estimated maximum tumor volume of 1,687.5 mm^3^ when calculated using the above formula. Mice were monitored regularly for tumor burden, body weight, general appearance, mobility, food and water intake, and signs of pain or distress. Animals were euthanized if they showed severe distress, marked weight loss, impaired mobility, ulceration or necrosis of the tumor, inability to access food or water, or if the maximum tumor diameter approached or exceeded 15 mm. Animal health and behavior were monitored at least once daily, and tumor growth was measured regularly during the experimental period. All efforts were made to minimize animal suffering and distress, including controlled housing conditions, careful handling, anesthesia before euthanasia, and timely euthanasia when humane endpoints were reached.

After 7 weeks, all nude mice were deeply anesthetized by intraperitoneal injection of sodium pentobarbital at 150 mg/kg. Following confirmation of deep anesthesia, as indicated by the absence of pedal and corneal reflexes, euthanasia was performed by cervical dislocation. Tumors were then removed, weighed, and photographed. A total of 25 mice were used in the subcutaneous xenograft experiment. All animals were euthanized at the planned experimental endpoint or earlier if humane endpoints were reached, and no animals were found dead during the study.

For the orthotopic liver xenograft model, the cell suspension was adjusted to a density of 1 × 10^6^ cells and administered via intrahepatic injection in nude mice. A total of 25 mice were used in the orthotopic liver xenograft model and randomly assigned to 5 groups, with 5 mice in each group. The experiment lasted for 2 weeks. Liver metastatic lesions were subsequently assessed using an in vivo bioluminescence imaging system (IVIS® Lumina III). Mice in this model were also monitored regularly for body weight, activity, posture, food and water intake, abdominal distension, and other signs of pain or distress. Humane endpoints were applied as described above. All mice in the orthotopic liver xenograft model were euthanized at the planned experimental endpoint or earlier if humane endpoints were reached, and no animals were found dead during the study.The sample size was determined based on previous studies using comparable HCC xenograft models and practical considerations; no formal a priori power analysis was performed. After tumor establishment, mice were randomly assigned to the indicated experimental groups using a random number table. Investigators responsible for tumor measurement and outcome assessment were blinded to group allocation. No animals were excluded from the analysis unless predefined humane endpoint criteria were met.

All animal procedures were carried out in strict accordance with the ethical guidelines for animal experimentation of Guizhou Medical University. The animal experimental protocol was approved by the Laboratory Animal Ethics Committee of Guizhou Medical University, approval No. 2601004. All procedures were performed with efforts to minimize animal suffering.

### 2.20 Construction of shRNA-resistant ENO2 wild-type and catalytic-deficient mutant constructs and rescue experiments

To determine whether ENO2 catalytic activity contributes to ENO2-associated phenotypes, shRNA-resistant ENO2 wild-type (ENO2-WT) and shRNA-resistant catalytic-deficient ENO2 mutant (ENO2-E211Q) constructs were generated in the pcDNA3.1 expression vector. The empty pcDNA3.1 vector was used as the corresponding control. ENO2 shRNA was cloned into the pLKO vector. Silent nucleotide substitutions were introduced into the shRNA-targeting region of the ENO2 coding sequence without altering the amino acid sequence, thereby preventing recognition by ENO2 shRNA. The catalytic-deficient mutant was generated by site-directed mutagenesis to introduce the E211Q substitution. All plasmids were confirmed by Sanger sequencing.

HCC cells were first transduced with pLKO-based ENO2 shRNA or control shRNA and subsequently transfected with shRNA-resistant pcDNA3.1-ENO2-WT, pcDNA3.1-ENO2-E211Q, or empty pcDNA3.1. Re-expression of ENO2 was verified by western blotting. Cells were then subjected to the indicated assays, including ECAR, glucose uptake, lactate production, ATP measurement, cell viability, colony formation, migration, and invasion.

### 2.21 Glycolytic inhibition and pyruvate rescue experiments

To determine whether glycolytic activity contributes to ENO2-associated phenotypes, ENO2-overexpressing HCC cells were treated with 2-deoxy-D-glucose (2-DG; MCE,HY-13966), a glycolysis inhibitor, at 3mM for 24h. Vehicle-treated cells were used as controls. In parallel, ENO2-knockdown cells were supplemented with sodium pyruvate (solarbio, P9810) at 1mM for 24h to evaluate metabolic rescue.Cells were subsequently subjected to the indicated metabolic, proliferation, colony-formation, migration, invasion, and signaling assays.

### 2.22 Statistical analysis

Statistical analyses were performed using R 4.2.1 and GraphPad Prism 9.0. Data are presented as mean ± SD unless otherwise stated. Student’s t-test, one-way ANOVA with post hoc tests, or nonparametric tests were used as appropriate. Correlations were assessed using Pearson correlation analysis. Survival analyses were performed using the Kaplan–Meier method and log-rank tests. Pan-cancer prognostic effects were evaluated using univariate Cox regression. ROC analysis was used to calculate AUC values. All in vitro experiments were independently repeated at least three times. A value of *P < 0.05 was considered statistically significant. Statistical significance levels are indicated as follows:*P < 0.05, **P < 0.01, ***P < 0.001, ****P < 0.0001;ns, not significant.

## 3 Results

### 3.1 Identification of glucose metabolism-related candidate genes in HCC and pan-cancer analysis

To identify HCC-associated genes, three GEO datasets, GSE45267, GSE101685, and GSE84402, were integrated after batch-effect correction. Box plots, UMAP analysis, and density plots showed improved comparability of gene expression distributions across datasets after correction and normalization (Fig 1A–F).

**Fig 1:**
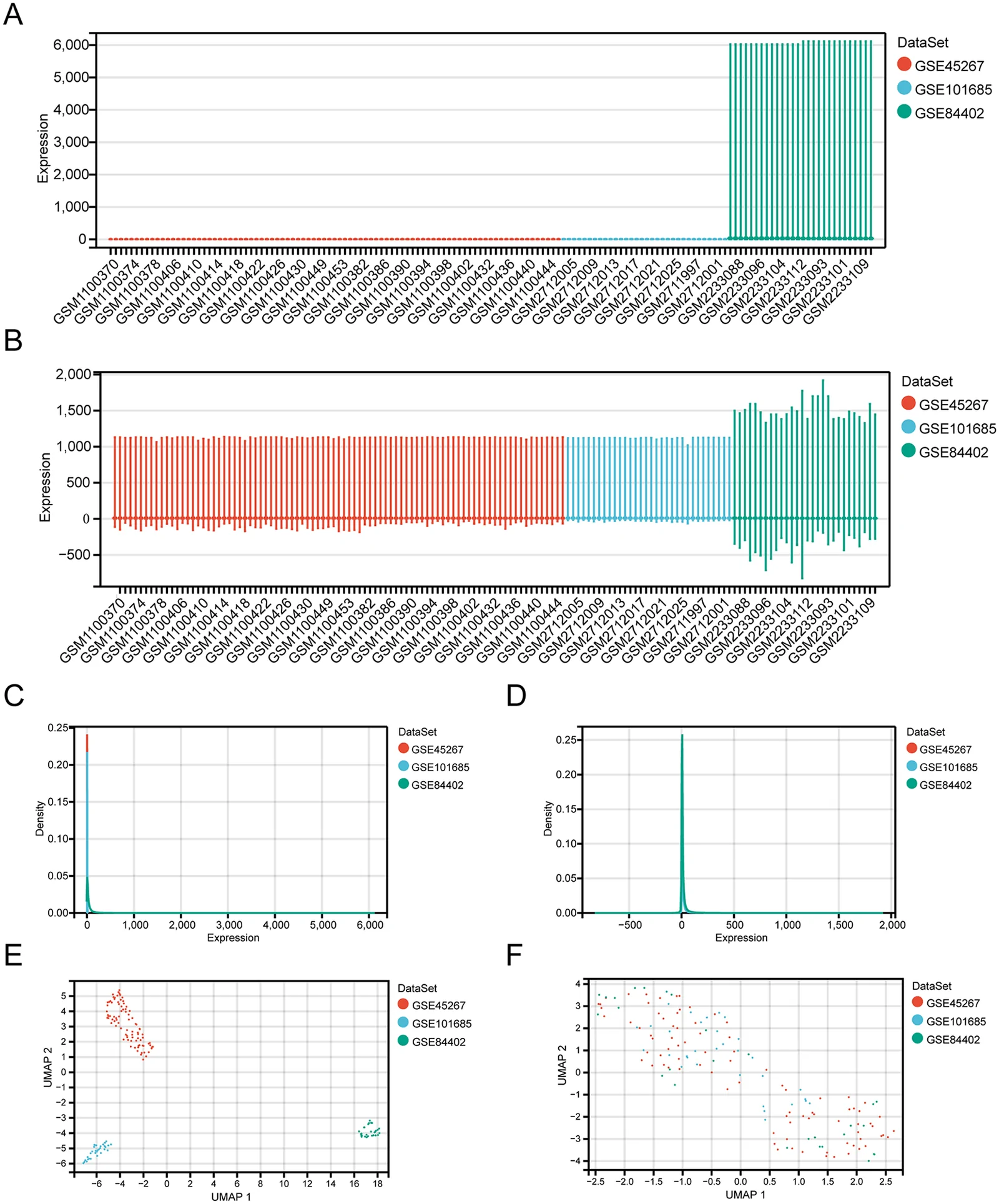
Integration of datasets and batch effect correction. (A–B) Boxplots showing normalization of gene expression distributions across datasets before and after correction. (C–D) UMAP plots demonstrating effective integration of samples without dataset-specific clustering. (E–F) Density plots indicating consistent expression distributions after batch effect removal. Data are presented as mean ± SD.*P < 0.05, **P < 0.01, ***P < 0.001, ****P < 0.0001;ns, not significant.

Weighted gene co-expression network analysis was then performed using the merged expression matrix. The soft-thresholding power was set at β = 5, with a scale-free topology index of 0.86 (Fig 2A–B). Hierarchical clustering identified 29 co-expression modules using a minimum module size of 30 and a module merging threshold of 0.25 (Fig 2C–D).the Brown module exhibited the strongest positive correlation with tumor status,with a correlation coefficient of 0.88 (P = P = 5.0e-49; Fig 2E). Within the Brown module, gene significance (GS) for tumor status was strongly positively correlated with module membership (MM) and contained 143 candidate genes (r = 0.96, P < 2.2e-16; Fig 2F)

**Fig 2:**
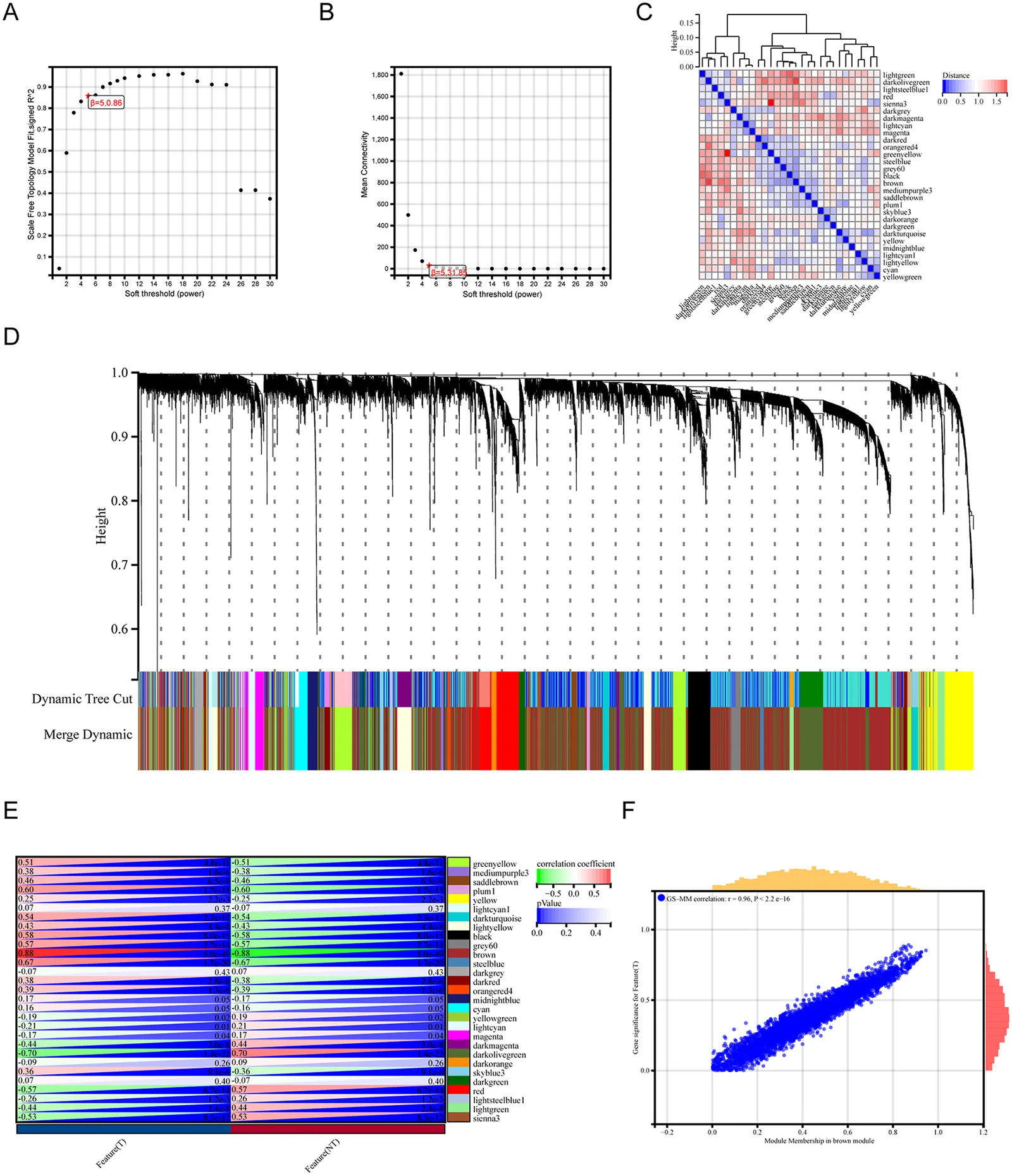
WGCNA identifies tumor-associated gene modules. (A-B) Determination of the optimal soft-thresholding power (β = 5) based on scale-free topology and mean connectivity. (C-D) Hierarchical clustering dendrogram and module identification using dynamic tree cutting and module merging. (E) Module–trait correlation heatmap showing the correlations between module eigengenes and tumor or normal status. The color intensity indicates the correlation coefficient, and the numbers in each cell indicate the corresponding correlation coefficient and P-value. The Brown module showed the strongest positive correlation with tumor status (r = 0.88, P = 5.0e-49).T, tumor; N, normal. (F) Gene significance/module membership correlation plot for genes in the Brown module. A strong positive correlation was observed between gene significance for tumor status and module membership (r = 0.96, P < 2.2e-16). GS, gene significance; MM, module membership.

As an exploratory transcriptomic screen, RNA-seq was performed using three paired HCC and adjacent non-tumor tissues. Given the limited sample size, these results were integrated with WGCNA-derived genes and glycolysis-related gene sets for target selection. A total of 1,968 differentially expressed genes were identified in HCC tissues, including 1,068 upregulated and 900 downregulated genes (|log2FC| > 1, P < 0.05; Fig 3A–B). This intersection prioritized ENO2 as a candidate gene for downstream validation rather than establishing it as a definitive driver of HCC. The selection of ENO2 was subsequently supported by analyses of independent public datasets and functional experiments.Enrichment analyses showed that these genes were mainly involved in small-molecule metabolism, wound healing, glucose metabolism/glycolysis, pyruvate metabolism, central carbon metabolism in cancer, and the tricarboxylic acid cycle (Fig 3C). GSEA further linked the differentially expressed genes to p53, PI3K, MAPK, Wnt, cell-cycle, liver cancer, hepatitis B-related, and glucose metabolism pathways (Fig 3D–L).

**Fig 3:**
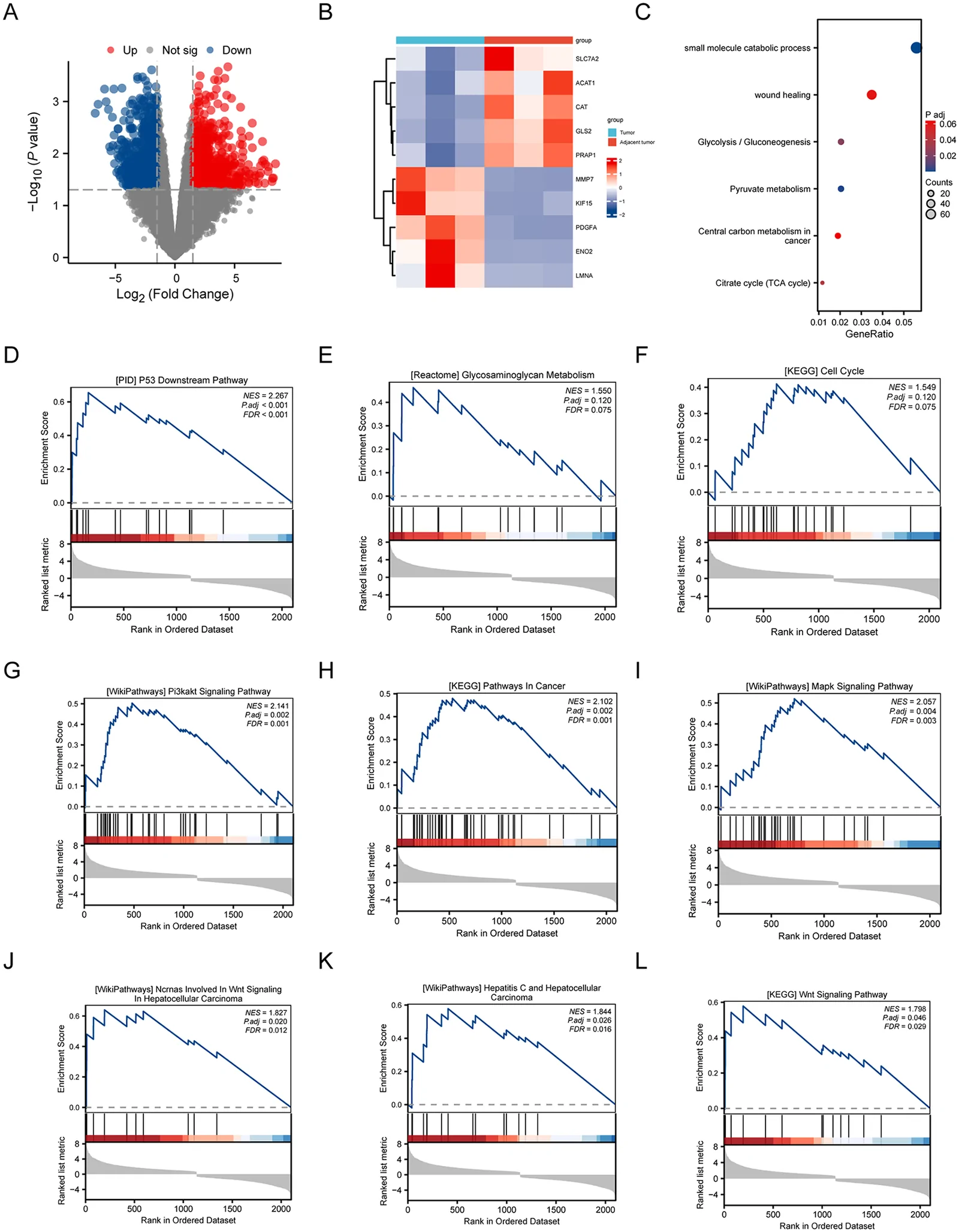
RNA-seq identifies differentially expressed genes and enriched pathways in HCC. (A-B) Volcano plot and heatmap showing differentially expressed genes (DEGs) between HCC and adjacent normal tissues. (C) Functional enrichment analysis indicates that DEGs are mainly involved in glycolysis, pyruvate metabolism, TCA cycle, and small molecule metabolism. (D-L) GSEA reveals enrichment of key signaling pathways, including P53, PI3K, MAPK, Wnt signaling, cell cycle, and glucose metabolism.

To narrow down glucose metabolism-related targets in HCC, 143 WGCNA candidate genes were intersected with 542 glucose metabolism-related genes from MSigDB and 486 upregulated genes from RNA-seq analysis (|log2FC| > 2, P < 0.05; Fig 4A–B). ENO2 was identified as a candidate glucose metabolism-related gene. ROC analysis showed modest diagnostic performance for HCC, with an AUC of 0.668 (Fig 4C). Pan-cancer analysis showed increased ENO2 expression in several tumors, including HCC, breast cancer, cholangiocarcinoma, and colon cancer (Fig 4D–E). ENO2 was also associated with poor prognosis in multiple cancers, including colon cancer, head and neck squamous cell carcinoma, renal chromophobe carcinoma, and HCC (Fig 4F).

**Fig 4:**
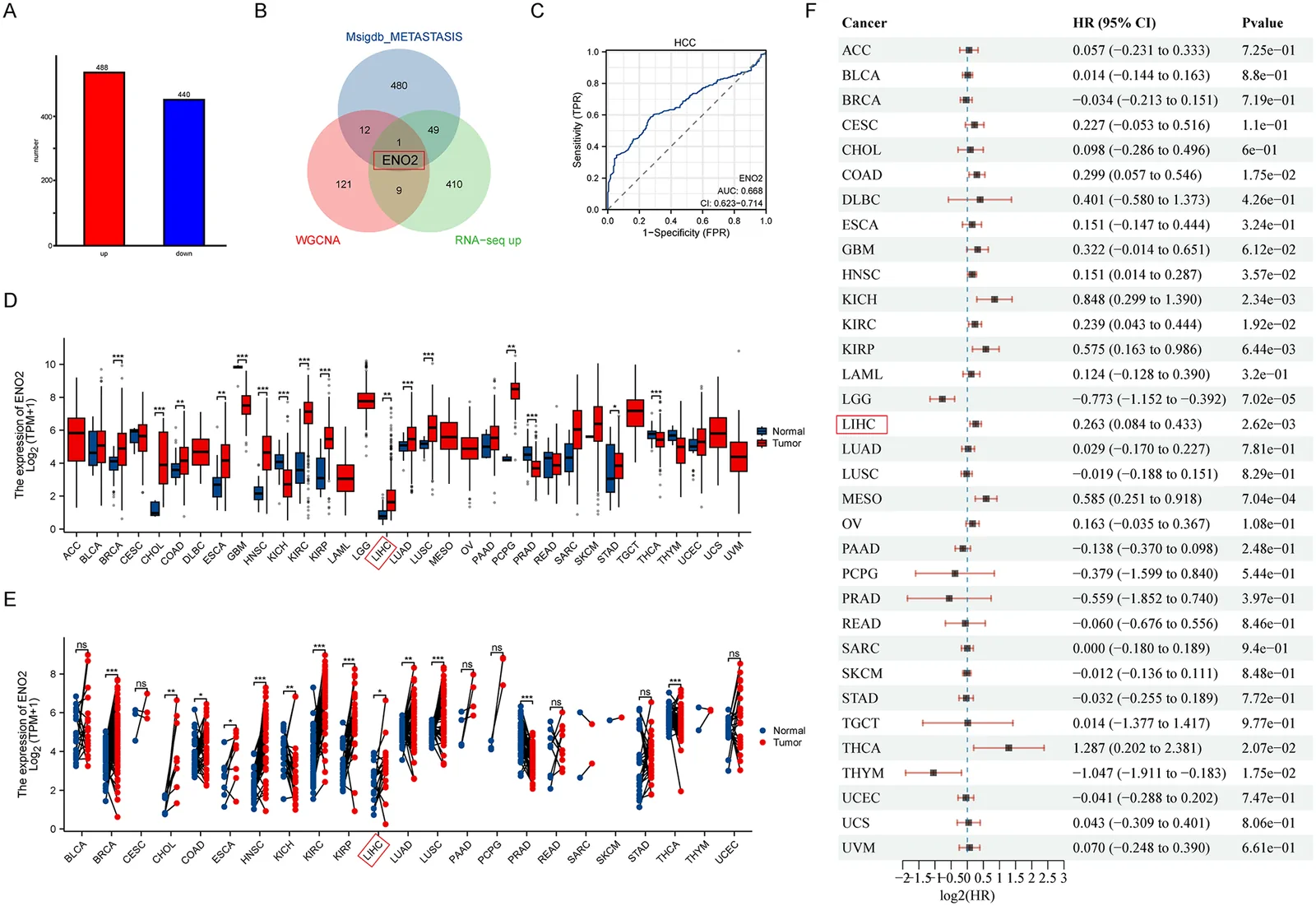
Identification of ENO2 as a key glycolysis-related gene in HCC. (A-B) Venn diagram showing the overlap among WGCNA-derived hub genes, upregulated DEGs, and glycolysis-related genes from MSigDB. (C) ROC curve evaluating the diagnostic performance of ENO2 in HCC. (D-E) Pan-cancer analysis showing ENO2 expression across multiple tumor types. Forest plot showing the prognostic significance of ENO2 in different cancers. Data are presented as mean ± SD.*P < 0.05, **P < 0.01, ***P < 0.001, ****P < 0.0001;ns, not significant.

### 3.2 Bioinformatic analysis of ENO2 in HCC

Analysis of TCGA and GTEx data showed higher ENO2 expression in HCC tissues than in normal liver tissues (Fig 5A). Consistent with this finding, Human Protein Atlas data indicated increased ENO2 protein expression in HCC tissues (Fig 5B). Clinicopathological analysis showed that ENO2 expression was positively associated with tumor stage (Fig 5C), with higher levels in T3–T4 tumors than in T1–T2 tumors (Fig 5D). Higher ENO2 expression was also associated with lymphatic metastasis (Fig 5E). Survival analysis showed that patients with high ENO2 expression had shorter overall survival(OS), disease-specific survival(DSS), and progression-free interval(PFI) (Fig 5F–H).

**Fig 5:**
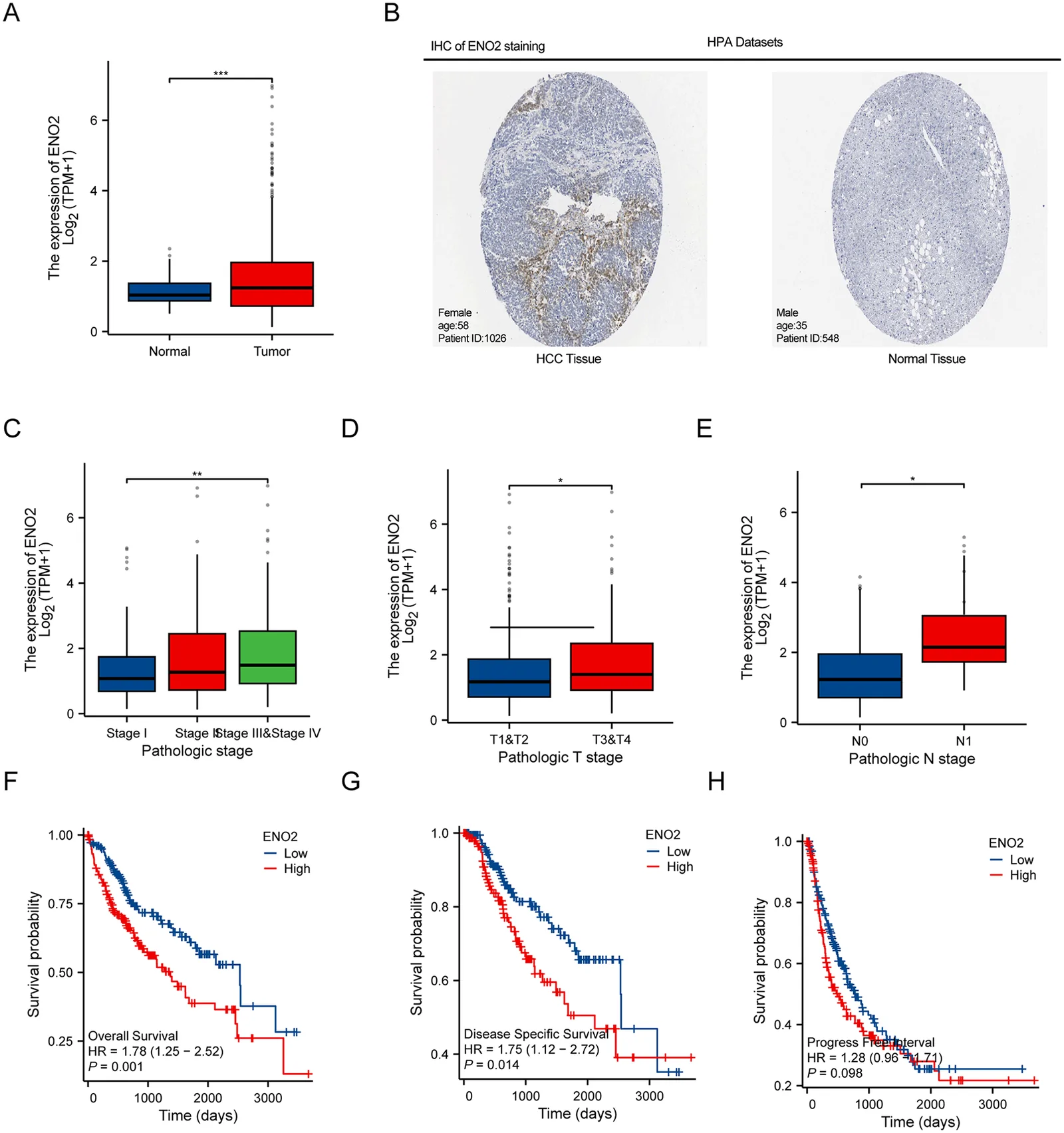
Clinical significance of ENO2 in HCC. (A) ENO2 expression in TCGA-LIHC dataset. (B) Protein expression of ENO2 in HCC tissues from the HPA database. (C-E) Association between ENO2 expression and clinicopathological features, including pathological stage, T stage, and lymph node metastasis. (F-H) Kaplan–Meier survival curves showing OS, DSS, and PFI stratified by ENO2 expression. Data are presented as mean ± SD.*P < 0.05, **P < 0.01, ***P < 0.001, ****P < 0.0001;ns, not significant.

To explore ENO2-related biological processes, ssGSEA was performed in the TCGA-HCC cohort. ENO2 expression was positively associated with several cancer-related pathways and phenotypes, including PI3K/AKT, TGF-β, MYC signaling, proliferation, DNA replication, angiogenesis, inflammatory response, EMT, and extracellular matrix degradation (Fig 6A–I). These findings suggest that ENO2 is associated with aggressive biological features in HCC.

**Fig 6:**
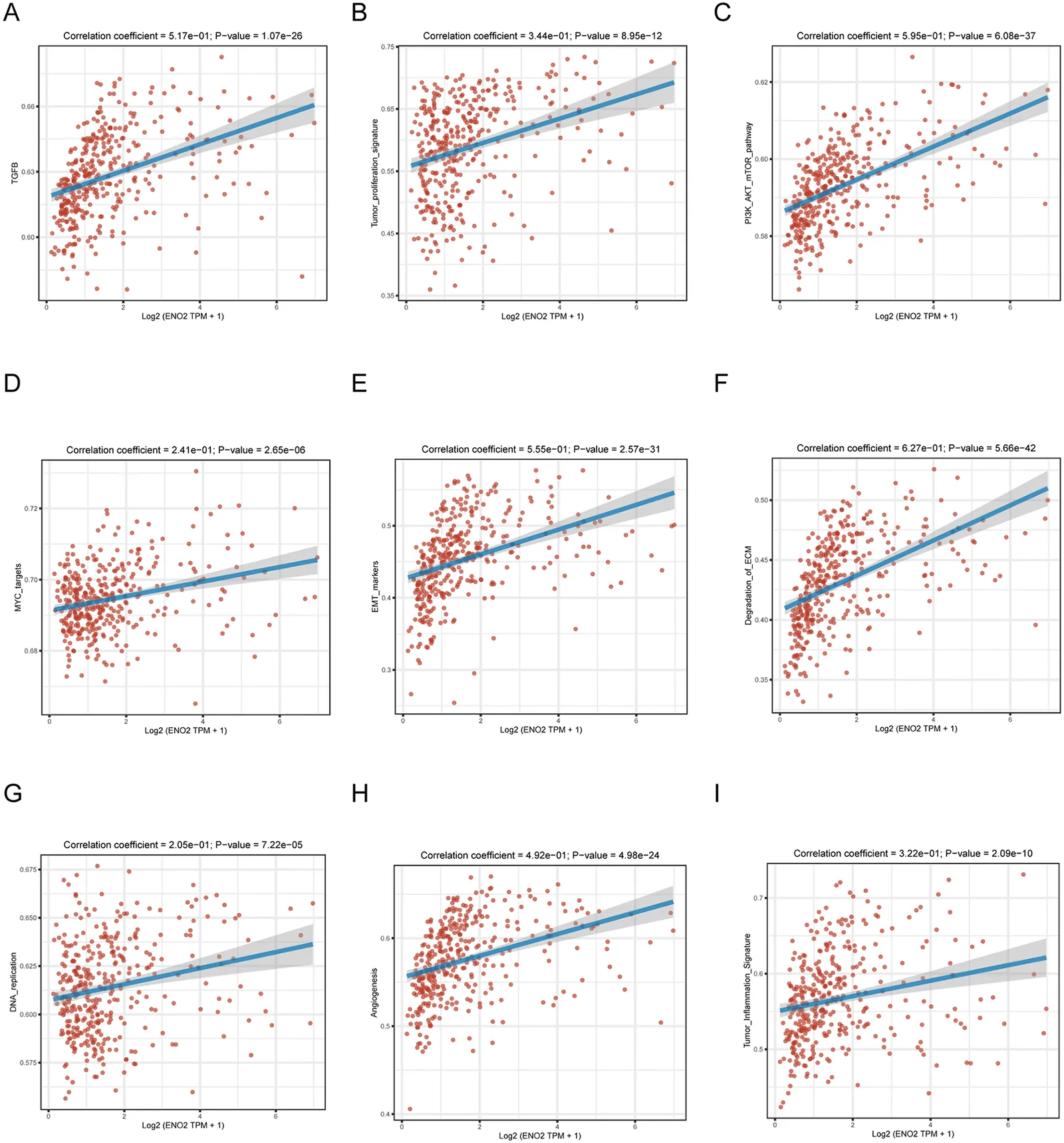
Functional enrichment analysis of ENO2. (A-I) ssGSEA results showing that ENO2 is associated with tumor-related biological processes, including proliferation, EMT, ECM degradation, angiogenesis, inflammation, and key signaling pathways such as PI3K/AKT, TGF-β, and MYC.

### 3.3 ENO2 is upregulated in HCC cells and tissues

To validate ENO2 expression in HCC, we examined several HCC cell lines and the authenticated normal hepatocyte line THLE2. qRT-PCR showed higher ENO2 mRNA expression in most HCC cell lines than in THLE2 cells (Fig 7A). Western blotting and densitometric analysis showed a similar increase at the protein level (Fig 7B–C). Based on ENO2 expression patterns and experimental suitability, SNU398 and LM3 cells were selected for subsequent gain- and loss-of-function assays.

**Fig 7:**
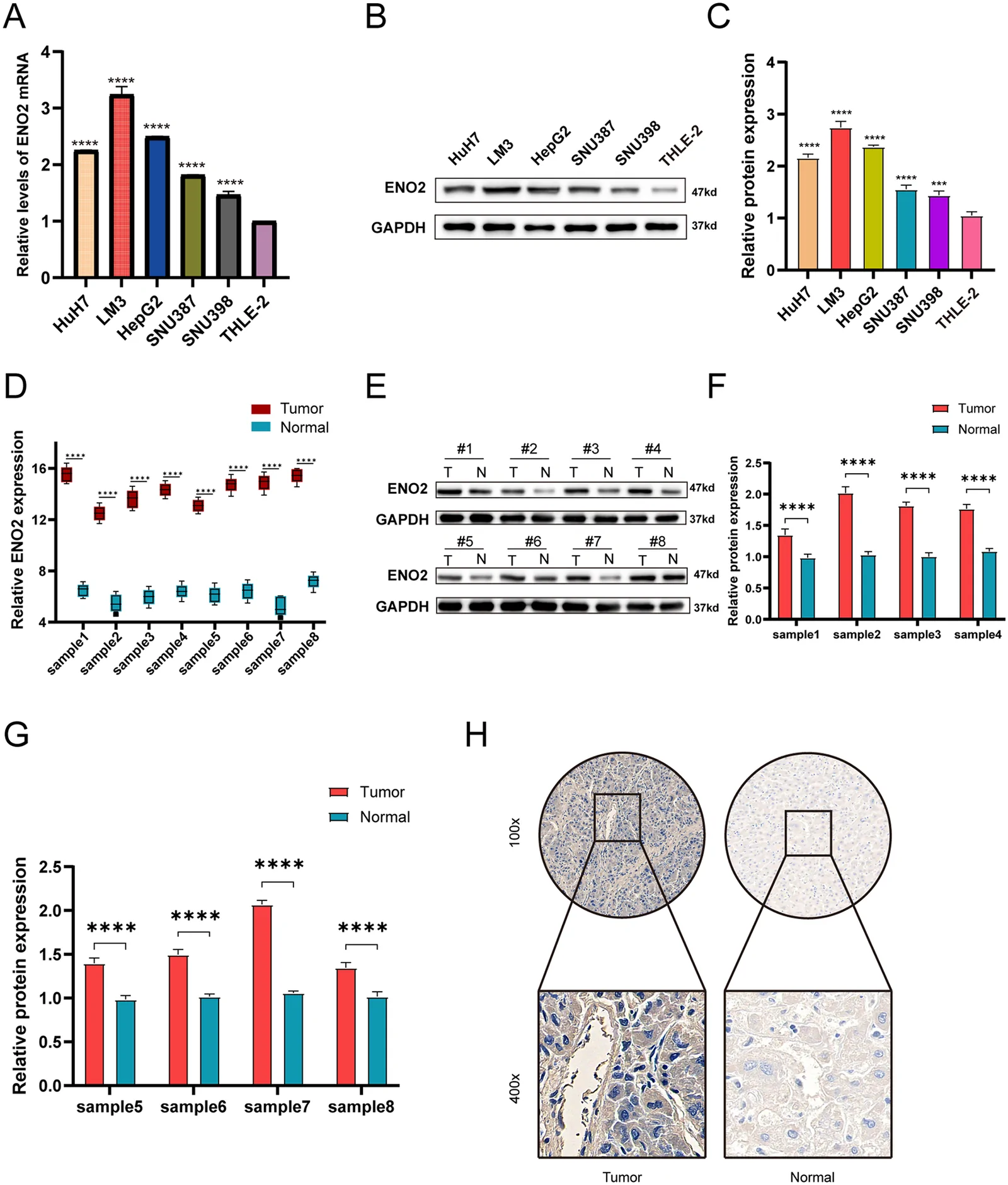
Validation of ENO2 expression in HCC cell lines and clinical tissues. (A) ENO2 mRNA expression levels in HCC cell lines and the the authenticated normal hepatocyte line THLE2. (B) Representative Western blot images showing ENO2 protein expression in HCC cell lines and THLE2 cells. (C) Densitometric quantification of ENO2 protein expression shown in panel B. (D) ENO2 mRNA expression in eight paired HCC tissues and adjacent non-tumor tissues. (E) Representative Western blot images showing ENO2 protein expression in eight paired HCC tissues and adjacent non-tumor tissues. T, tumor tissue; N, adjacent non-tumor tissue. (F–G) Densitometric quantification of ENO2 protein expression in paired HCC and adjacent non-tumor tissues shown in panel E, including samples 1–4 (F) and samples 5–8 (G). (H) Representative immunohistochemical staining images showing ENO2 expression in HCC tissues and adjacent non-tumor tissues. Magnification: 100× and 400 × . Data are presented as mean ± SD from three independent experiments.*P < 0.05, **P < 0.01, ***P < 0.001, ****P < 0.0001;ns, not significant.

Public-dataset analyses showed that ENO2 expression was higher in HCC tissues than in non-tumor liver tissues and that high ENO2 expression was associated with shorter overall survival, disease-specific survival, and progression-free interval. In the in-house cohort of eight paired specimens, ENO2 mRNA levels were higher in HCC tissues than in matched adjacent tissues (Fig 7D). Western blotting confirmed increased ENO2 protein expression in HCC tissues, and densitometric quantification showed a consistent increase across paired samples (Fig 7E–G). Immunohistochemistry also showed stronger ENO2 staining in HCC tissues than in adjacent non-tumor tissues (Fig 7H). Because this small cohort lacked complete clinicopathological and follow-up data, it was used exclusively for expression validation and did not provide independent prognostic validation.These results indicate that ENO2 is upregulated in HCC cells and clinical specimens.

### 3.4 ENO2 promotes HCC cell proliferation, migration, invasion, and tumor growth

To investigate the function of ENO2 in HCC, three ENO2-targeting siRNAs were screened. si-ENO2#2 and si-ENO2#3 showed the most efficient knockdown and were used in subsequent experiments (Fig 8A-B). Stable ENO2-overexpression and ENO2-knockdown cell models were then established using lentiviral vectors, and transfection efficiency was confirmed (Fig 8C-D).

**Fig 8:**
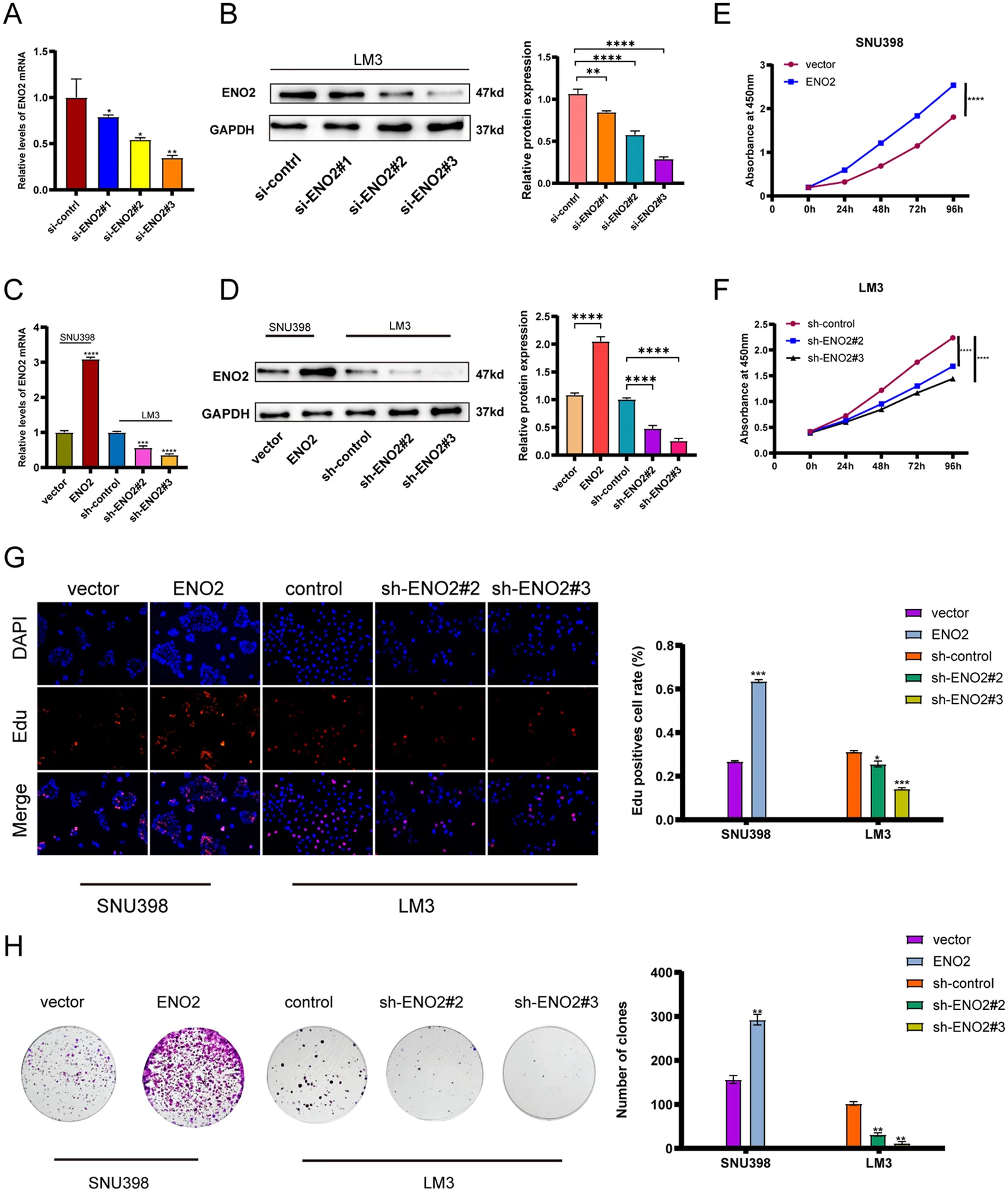
ENO2 promotes HCC cell proliferation. (A) Validation of siRNA-mediated ENO2 knockdown efficiency in LM3 cells by qRT-PCR. (B) Representative Western blot images and densitometric quantification showing siRNA-mediated ENO2 knockdown efficiency in LM3 cells. (C) Validation of ENO2 overexpression in SNU398 cells and shRNA-mediated ENO2 knockdown in LM3 cells by qRT-PCR. (D) Representative Western blot images and densitometric quantification showing ENO2 overexpression and knockdown efficiency in HCC cells. (E) CCK-8 assay showing that ENO2 overexpression promotes the proliferation of SNU398 cells. (F) CCK-8 assay showing that ENO2 knockdown suppresses the proliferation of LM3 cells. (G) Representative images and quantification of EdU staining showing the effects of ENO2 overexpression or knockdown on HCC cell proliferation. (H) Representative images and quantification of colony formation assays showing the effects of ENO2 overexpression or knockdown on the clonogenic ability of HCC cells. Data are presented as mean ± SD from three independent experiments.*P < 0.05, **P < 0.01, ***P < 0.001, ****P < 0.0001;ns, not significant.

CCK-8, EdU, and colony formation assays showed that ENO2 overexpression increased HCC cell proliferation, whereas ENO2 knockdown reduced proliferative capacity (Fig 8E–H). Transwell assays showed that ENO2 overexpression enhanced cell migration and invasion, while ENO2 knockdown suppressed these abilities (Fig 9A). Wound-healing assays produced consistent results, with faster wound closure in ENO2-overexpressing cells and delayed migration after ENO2 knockdown (Fig 9B).

**Fig 9:**
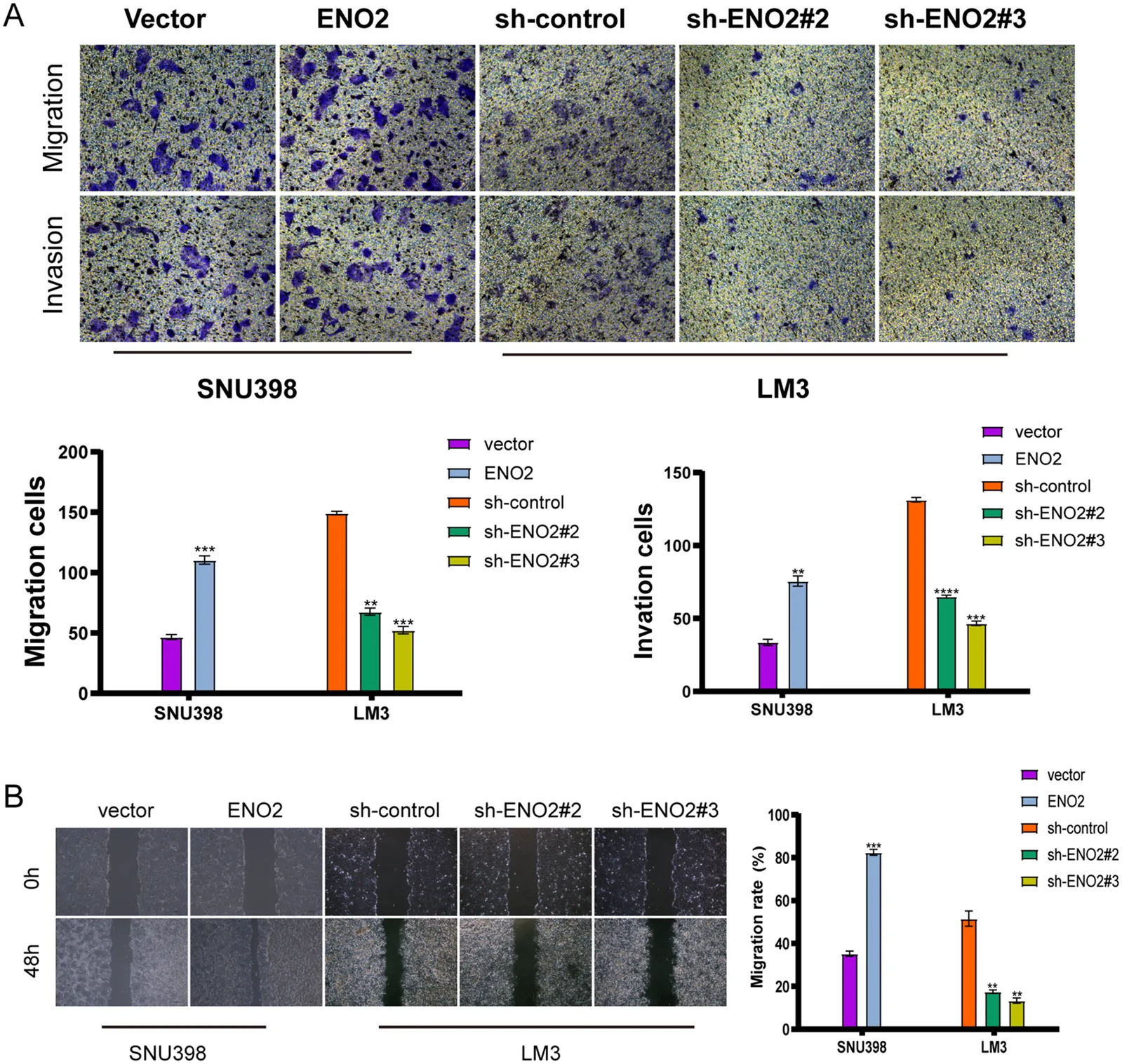
ENO2 enhances migration and invasion of HCC cells. (A) Representative images and quantitative analysis of Transwell migration and Matrigel invasion assays in SNU398 and LM3 cells with ENO2 overexpression or knockdown. ENO2 overexpression increased the number of migrated and invaded cells, whereas ENO2 knockdown markedly reduced cell migration and invasion. (B) Representative images and quantitative analysis of wound-healing assays showing the migratory capacity of SNU398 and LM3 cells at 0, 24, and 48 h after scratching. ENO2 overexpression accelerated wound closure, while ENO2 knockdown delayed wound closure. Data are presented as mean ± SD from at least three independent experiments.*P < 0.05, **P < 0.01, ***P < 0.001, ****P < 0.0001;ns, not significant.

The role of ENO2 was further evaluated in vivo using subcutaneous xenograft and orthotopic liver tumor models. In the subcutaneous model, tumors derived from ENO2-overexpressing cells were larger than control tumors, whereas ENO2 knockdown reduced tumor size (Fig 10A). Tumor weight and growth curves confirmed that ENO2 overexpression promoted tumor growth, while ENO2 silencing suppressed tumor progression (Fig 10B–C). The largest tumor volume recorded in the subcutaneous xenograft experiment was 709.8 mm^3^, and no tumor exceeded the predefined humane endpoint criterion of a maximum tumor diameter approaching or exceeding 15 mm. In the orthotopic liver tumor model, representative bioluminescence images and quantitative signal analysis showed increased tumor burden in the ENO2-overexpression group and reduced tumor progression after ENO2 knockdown (Fig 10D–E).Together, these findings indicate that ENO2 promotes HCC cell proliferation, migration, invasion, and tumor growth.

**Fig 10:**
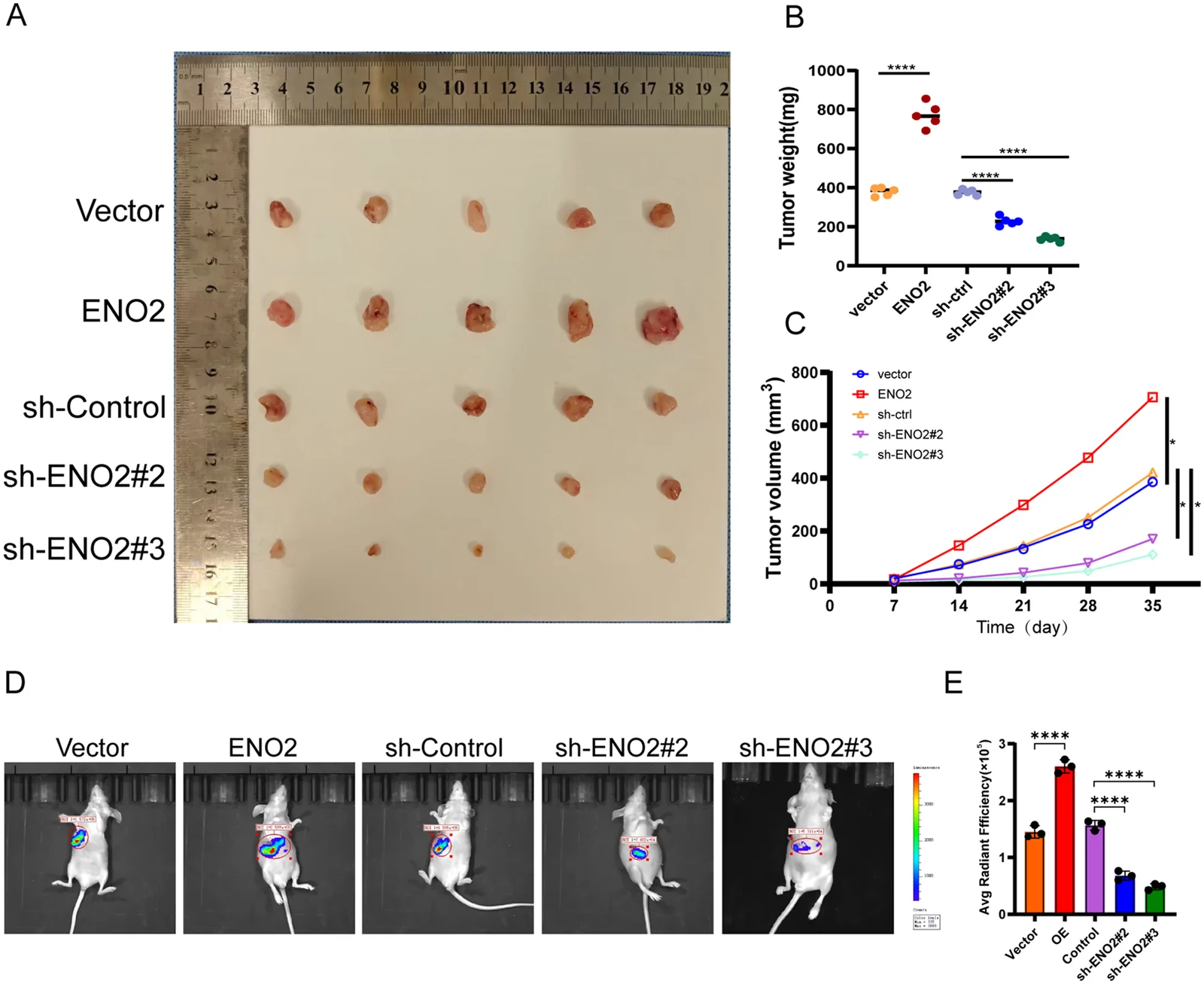
ENO2 promotes tumor growth and metastasis in vivo. (A) Representative images of subcutaneous xenograft tumors derived from HCC cells with ENO2 overexpression or knockdown. (B) Tumor weight analysis showing that ENO2 overexpression significantly increases tumor mass, whereas ENO2 knockdown reduces tumor weight. (C) Tumor growth curves indicating that ENO2 overexpression accelerates tumor progression, while ENO2 knockdown suppresses tumor growth. (D) Representative bioluminescence images from the orthotopic liver tumor model. (E) Quantitative analysis of bioluminescence intensity demonstrating that ENO2 overexpression enhances tumor burden, whereas ENO2 knockdown markedly inhibits tumor progression. All images and data shown in this figure were generated by the authors for the present study. Data are presented as mean ± SD from at least three independent experiments.*P < 0.05, **P < 0.01, ***P < 0.001, ****P < 0.0001;ns, not significant.

### 3.5 ENO2 enhances glycolytic metabolism in HCC cells

To determine whether ENO2 affects glucose metabolism in HCC cells, lactate production, glucose consumption, ATP levels, and extracellular acidification rate were examined after ENO2 modulation. ENO2 overexpression increased lactate production, whereas ENO2 knockdown reduced lactate levels (Fig 11A). Glucose consumption, estimated by measuring residual glucose in the culture medium, was increased in ENO2-overexpressing cells and decreased after ENO2 knockdown (Fig 11B). ATP levels showed a similar pattern, with increased ATP production after ENO2 overexpression and reduced ATP levels after ENO2 depletion (Fig 11C).

**Fig 11:**
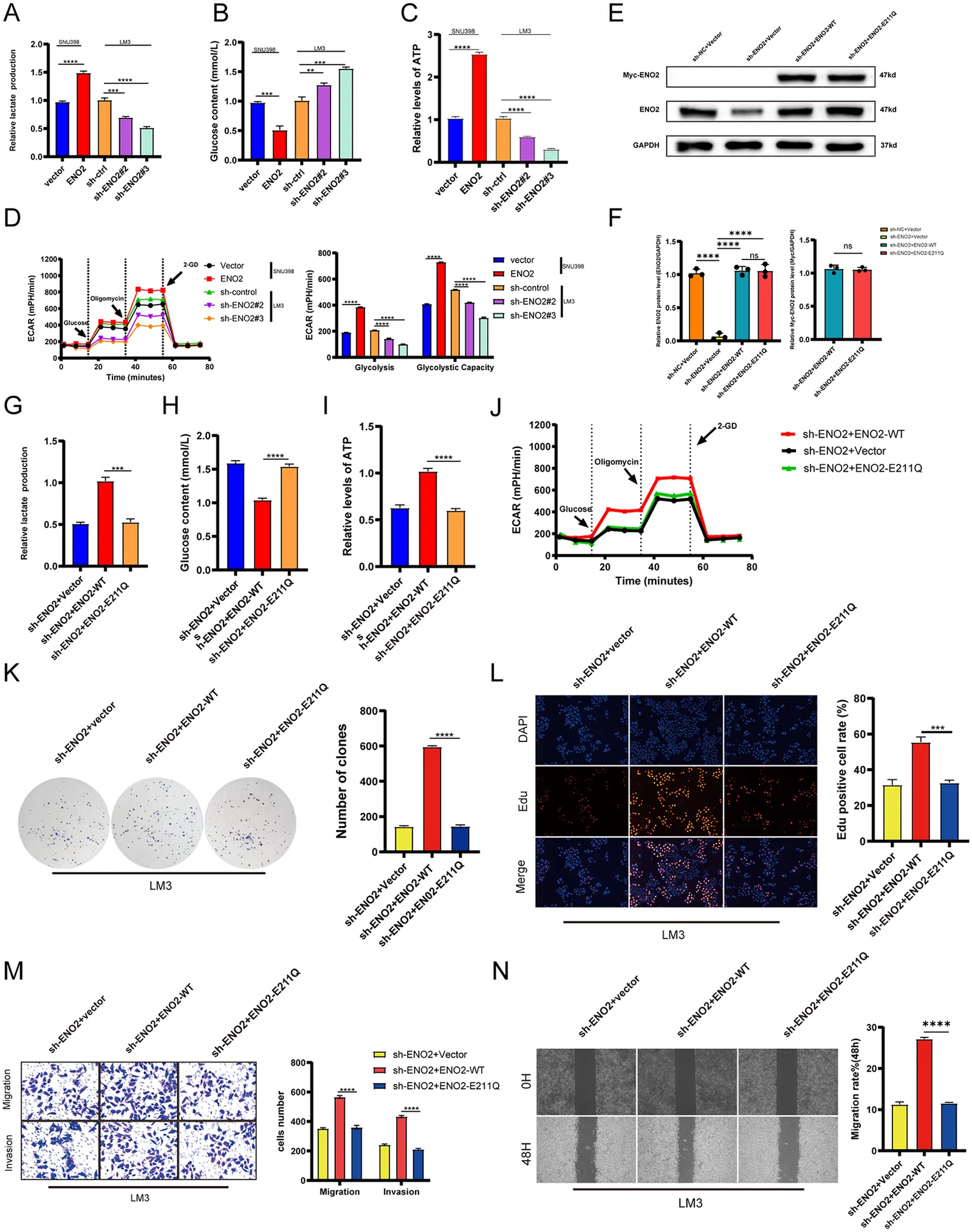
ENO2 regulates glycolytic metabolism in HCC cells. (A) Measurement of lactate production, showing that ENO2 overexpression significantly increases lactate levels, whereas ENO2 knockdown reduces lactate production. (B) Glucose content in the culture medium, indicating higher glucose consumption in ENO2-overexpressing cells and lower consumption in ENO2-knockdown cells. (C) Intracellular ATP level analysis, demonstrating that ENO2 overexpression enhancesATP production, whereas ENO2 knockdown reducesATPlevels. ENO2 enhances glycolytic capacity in HCC cells. (D) Seahorse XFglycolysis stress test measuring glycolysis function in SNU398 and LM3 cells. Glucose, oligomycin, and 2-DG were sequentially added, and ECAR (mpH/min) was recorded. ENO2 overexpression significantly increased glycolysis, while ENO2 knockdownsuppressed glycolytic capacity. (E) Representative Western blots showing total ENO2, exogenous MYC-tagged ENO2-WT or ENO2-E211Q, and the corresponding loading control in ENO2-depleted HCC cells. (F) Densitometric quantification of total ENO2 and exogenous MYC-tagged ENO2-WT or ENO2-E211Q protein expression normalized to the corresponding loading control. (G–I) Relative lactate production, glucose consumption, and intracellular ATP levels in LM3 cells with ENO2 knockdown and re-expression of shRNA-resistant ENO2-WT or catalytic-deficient ENO2-E211Q. (J) Real-time extracellular acidification rate (ECAR) analyzed using a Seahorse extracellular flux analyzer. The indicated sequential injections of glucose, oligomycin, and 2-DG are shown. (K) Representative images and quantification of colony-formation assays. (L) Representative images and quantification of EdU incorporation assays. DAPI, blue; EdU, red; merged images are shown. (M) Representative images and quantification of Transwell migration and invasion assays. (N) Representative images and quantification of wound-healing assays at 0 and 48 h. Data are presented as mean ± SD from at least three independent experiments.*P < 0.05, **P < 0.01, ***P < 0.001, ****P < 0.0001;ns, not significant.

The Seahorse XF Glycolysis Stress Test further showed that ENO2 altered glycolytic activity in LM3 and SNU398 cells. After glucose addition, ENO2-overexpressing cells showed higher ECAR than control cells, whereas ENO2 knockdown reduced ECAR. Following oligomycin treatment, glycolytic capacity remained higher in ENO2-overexpressing cells and was impaired after ENO2 knockdown. Addition of 2-DG rapidly reduced ECAR to baseline levels in all groups (Fig 11D). These results suggest that ENO2 enhances glycolytic metabolism in HCC cells.

### 3.6 ENO2 enzymatic activity is required for ENO2-associated glycolytic and malignant phenotypes in HCC cells

To determine whether ENO2 catalytic activity contributes to ENO2-associated phenotypes, ENO2-depleted HCC cells were reconstituted with shRNA-resistant ENO2-WT or shRNA-resistant catalytic-deficient ENO2-E211Q.Re-expression of ENO2-WT restored the reductions in ECAR, glucose uptake, lactate production, and intracellular ATP levels induced by ENO2 knockdown. In contrast, ENO2-Mut failed to restore these glycolytic readouts (Fig 12A–D). Similarly, ENO2-WT, but not ENO2-Mut, rescued the impaired cell viability, colony formation, migration, and invasion caused by ENO2 depletion (Fig 12F–J). Together, these findings indicate that ENO2 enzymatic activity is required for, or makes a major contribution to, ENO2-associated glycolytic remodeling and malignant phenotypes in HCC cells.

**Fig 12:**
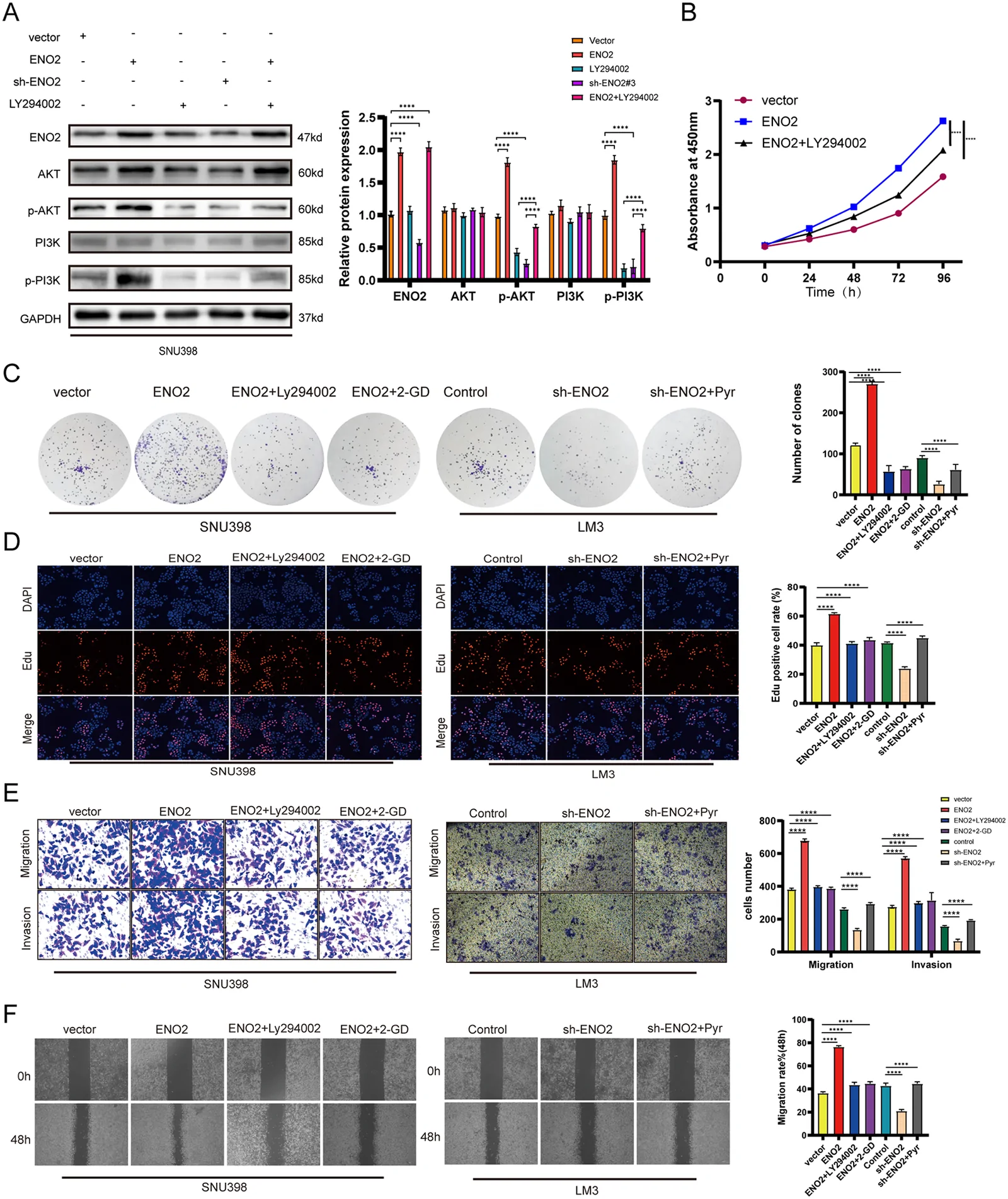
PI3K/AKT pathway inhibition and metabolic interventions attenuate ENO2-associated malignant phenotypes in HCC cells. (A) Representative Western blot images and densitometric quantification of PI3K/AKT pathway-related proteins in SNU398 cells. ENO2 overexpression increased the phosphorylation levels of PI3K and AKT without markedly altering total PI3K and AKT protein levels, whereas treatment with the PI3K inhibitor LY294002 reversed ENO2-induced PI3K/AKT activation. (B) CCK-8 assay showing that LY294002 attenuated the ENO2-induced increase in SNU398 cell viability. (C) Representative images and quantification of colony formation in SNU398 cells with ENO2 overexpression and the indicated treatments, and in LM3 cells with ENO2 knockdown with or without pyruvate supplementation. (D) Representative images and quantification of EdU incorporation in the indicated groups. (E) Representative images and quantification of Transwell migration and invasion assays in the indicated groups. (F) Representative images and quantification of wound-healing assays in the indicated groups. Data are presented as mean ± SD from three independent experiments.*P < 0.05, **P < 0.01, ***P < 0.001, ****P < 0.0001;ns, not significant.

### 3.7 PI3K/AKT pathway activity and glycolytic metabolism contribute to ENO2-associated malignant phenotypes in HCC cells

Enrichment analysis suggested a potential association between ENO2 and the PI3K/AKT signaling pathway. We therefore examined whether ENO2 expression was associated with PI3K/AKT pathway activity in HCC cells. Western blotting showed that ENO2 overexpression increased the phosphorylation levels of PI3K and AKT, whereas the total protein levels of PI3K and AKT remained largely unchanged. Treatment with the PI3K inhibitor LY294002 reduced PI3K and AKT phosphorylation in ENO2-overexpressing cells (Fig 12A).

We next evaluated the functional relevance of PI3K/AKT pathway activity. In SNU398 cells, ENO2 overexpression increased cell viability, colony-forming ability, DNA synthesis, migration, and invasion. These effects were attenuated by LY294002 treatment, as determined by CCK-8, colony formation, EdU incorporation, Transwell, and wound-healing assays (Fig 12B–F). These findings suggest that PI3K/AKT pathway activity contributes to the malignant phenotypes associated with ENO2 overexpression.

To further investigate the contribution of glycolytic metabolism, ENO2-overexpressing SNU398 cells were treated with the glycolysis inhibitor 2-DG. Compared with ENO2 overexpression alone, 2-DG treatment attenuated ENO2-associated increases in colony formation, EdU incorporation, migration, and invasion (Fig 12C–F). Conversely, pyruvate supplementation alleviated the reductions in colony-forming ability, DNA synthesis, migration, and invasion induced by ENO2 knockdown in LM3 cells (Fig 12C–F). Collectively, these results support that PI3K/AKT pathway activity and glycolytic metabolism contribute to ENO2-associated malignant phenotypes in HCC cells. Nevertheless, because LY294002 and 2-DG may exert effects beyond the individual pathways evaluated here, and pyruvate rescue was not uniformly complete across all assays, these data do not establish a direct regulatory relationship between ENO2 and PI3K/AKT signaling. The changes in PI3K/AKT phosphorylation may also be secondary to ENO2-associated metabolic alterations or other upstream regulatory mechanisms.

## 4 Discussion

HCC progression is closely linked to metabolic reprogramming and aberrant oncogenic signaling [20,21]. While previous studies have established ENO2 as a glycolysis-related gene in HCC through hypoxia-associated transcriptional regulation, non-coding RNA networks, and lactylation-associated feedback mechanisms, the present study provides a distinct integrated perspective. First, we prioritized ENO2 through the intersection of an HCC-associated WGCNA module, exploratory RNA-seq-derived candidates, and glycolysis-related gene sets, rather than selecting ENO2 from a single pre-specified regulatory pathway. Because RNA sequencing was performed in only three paired samples, it was used as an exploratory, hypothesis-generating component of this prioritization strategy and was interpreted together with WGCNA and independent public-dataset analyses. Second, we linked ENO2 perturbation to coordinated changes in glycolytic readouts and malignant phenotypes in HCC models, including subcutaneous xenograft model and orthotopic liver tumor model. Third, LY294002 experiments indicated that PI3K/AKT pathway activity contributes to ENO2-associated phenotypes. Collectively, these findings extend the current understanding of ENO2 biology in HCC beyond individual upstream regulatory axes.Importantly, the present study does not claim that ENO2 is newly identified as an HCC-associated glycolytic gene, nor does it establish direct molecular activation of PI3K/AKT signaling by ENO2. Instead, our data support that ENO2-associated metabolic remodeling is accompanied by altered PI3K/AKT phosphorylation and that inhibition of this pathway attenuates ENO2-associated phenotypes. The directionality and molecular intermediates underlying this relationship remain to be defined. This restrained interpretation distinguishes the present findings from mechanistic studies focused on specific ENO2 regulatory axes and identifies an integrated metabolism–signaling framework for future investigation.

The present study differs from previous ENO2 studies in HCC in both its analytical framework and experimental emphasis. Previous studies have predominantly investigated upstream mechanisms regulating ENO2 expression or activity. For example, hypoxia-related studies have examined HIF-1α-associated transcriptional regulation of ENO2, whereas studies involving the TUG1/miR-122-5p and circESYT2/miR-665 axes have focused on post-transcriptional regulation of ENO2 through non-coding RNA networks [22,23]. More recently, the H4K5 lactylation–ENO2 loop has been proposed as an epigenetic/metabolic feedback mechanism involving ENO2 [24]. In contrast, our study did not begin with a pre-specified upstream regulatory axis. Instead, ENO2 was prioritized through the intersection of an HCC-associated WGCNA module, differentially expressed genes identified by exploratory RNA sequencing, and glycolysis-related gene sets [25–30]. We subsequently evaluated the associations of ENO2 with glycolytic metabolism, malignant phenotypes, catalytic activity, and PI3K/AKT pathway-associated signaling. Thus, the contribution of this study is not the identification of ENO2 as a previously unknown HCC-associated glycolytic gene, nor the discovery of a novel upstream regulatory mechanism, but rather an integrated candidate-prioritization and functional-validation framework across metabolic, phenotypic, and signaling levels in HCC.

ENO2 is a glycolytic enzyme that catalyzes the conversion of 2-phosphoglycerate to phosphoenolpyruvate. Consistent with this biochemical role, ENO2 overexpression enhanced glycolytic activity, whereas ENO2 depletion produced the opposite effects. Rescue experiments further indicated that ENO2 catalytic activity makes an important contribution to these effects: shRNA-resistant ENO2-WT restored ENO2 activity and partially or substantially rescued glycolytic and malignant phenotypes in ENO2-depleted cells, whereas ENO2-E211Q failed to rescue, or showed substantially weaker rescue of, these phenotypes despite comparable expression of the exogenous MYC-tagged proteins. In addition, 2-DG attenuated the metabolic and malignant phenotypes associated with ENO2 overexpression, while pyruvate supplementation partially rescued the defects associated with ENO2 depletion. Collectively, these findings support a functional contribution of glycolytic metabolism to ENO2-associated malignant phenotypes. Nevertheless, because pyruvate rescue was incomplete and catalytic-site mutation may affect protein conformation, localization, stability, or protein interactions, the data do not exclude additional non-enzymatic functions of ENO2 or other downstream mechanisms.Besides,metabolic reprogramming in HCC is not limited to glycolysis but also involves lipid metabolism, redox homeostasis, and interactions with the tumor microenvironment [31,32]. In the present study, our data support a role for ENO2 in glycolytic reprogramming; however, its potential interactions with lipid metabolic pathways, oxidative stress, and the tumor microenvironment require further investigation.

HCC progression is driven by a complex and interconnected signaling network rather than a single pathway [33].We observed that ENO2 expression was associated with altered PI3K and AKT phosphorylation. LY294002 reduced PI3K/AKT phosphorylation and attenuated ENO2 overexpression-associated malignant phenotypes, suggesting that PI3K/AKT pathway activity contributes to these effects. However, Although PI3K/AKT signaling was examined in the present study because it was enriched in our transcriptomic analysis and is closely linked to glycolysis and malignant progression, other pathways may also contribute to ENO2-associated phenotypes. these experiments do not establish a direct molecular interaction between ENO2 and PI3K/AKT pathway components, nor do they determine the directionality of this relationship. other pathways may also contribute to ENO2-associated phenotypes; thus, PI3K/AKT should be considered a contributing rather than exclusive mediator.We assessed total and phosphorylated AKT but did not investigate the distinct roles of AKT1, AKT2, and AKT3, which is a limitation of this study. Future isoform-specific studies are needed.Moreover,the changes in PI3K/AKT phosphorylation may be secondary to ENO2-associated metabolic alterations, including altered glycolytic flux, ATP availability, lactate accumulation, redox status, or other upstream signaling events. Therefore, the present findings support an association between ENO2-related phenotypes and PI3K/AKT pathway activity, rather than direct activation of PI3K/AKT signaling by ENO2.

Recent studies have continued to identify candidate diagnostic biomarkers for HCC, including biomarkers associated with immune infiltration [34]. Nevertheless, diagnostic biomarkers require rigorous validation across independent cohorts and should demonstrate sufficient discriminatory performance before clinical application.The translational relevance of ENO2 should also be interpreted cautiously. Although high ENO2 expression was associated with worse survival outcomes in public datasets, the diagnostic performance of ENO2 alone was modest, with an AUC of 0.668. Therefore, ENO2 is unlikely to serve as a sufficiently accurate standalone diagnostic biomarker.In addition, the pan-cancer analysis indicated that the prognostic association of ENO2 was context-dependent, being favorable in some tumor types but unfavorable in others. These differences may reflect cancer-specific metabolic programs and molecular characteristics. Therefore, the biological and clinical relevance of ENO2 should be interpreted in a cancer-type-specific manner rather than as a uniform prognostic factor across malignancies.Future studies should evaluate whether ENO2 provides additional diagnostic or prognostic value when incorporated into multiparameter clinical, molecular, or metabolic models.

Despite these findings, several limitations of the present study should be acknowledged. First, the clinical cohort included only eight paired samples without complete clinicopathological and follow-up information; therefore, it was insufficient for clinicopathological correlation or independent survival validation. Second, RNA sequencing was performed in only three paired HCC and adjacent non-tumorous tissue samples. Although RNA sequencing was used as an exploratory component of the integrated screening strategy, its limited sample size reduces statistical power and increases the possibility of false-positive findings. Thus, these results should be considered preliminary and hypothesis-generating rather than definitive evidence for ENO2 selection. Third, although the comparable expression of exogenous Myc-tagged ENO2-WT and ENO2-E211Q supports the interpretation that the differential rescue effects were related to the E211Q mutation, catalytic-site mutation may still affect protein conformation, subcellular localization, or protein interactions. In addition, LY294002 experiments support the involvement of PI3K/AKT signaling but do not establish direct regulation by ENO2..Moreover, the 2-DG inhibition and pyruvate-rescue experiments do not establish that altered glycolysis is the sole mechanism underlying ENO2-associated phenotypes, and increased glycolytic activity may partly reflect the metabolic demands of enhanced cell proliferation. Larger clinically annotated cohorts, primary HCC cells, organoids, patient-derived models, genetic glycolysis-targeting approaches, and metabolic flux-tracing studies will be necessary to validate the findings and clarify how ENO2-associated metabolic remodeling intersects with PI3K/AKT signaling and other downstream mechanisms.

## 5 Conclusion

By integrating WGCNA, exploratory RNA sequencing, and glycolysis-related gene sets, this study prioritized ENO2 for functional investigation in HCC. ENO2 perturbation was associated with coordinated changes in glycolytic activity, malignant phenotypes, and PI3K/AKT phosphorylation. Pharmacological inhibition indicated that PI3K/AKT pathway activity contributes to ENO2-associated phenotypes, but direct activation of this pathway by ENO2 was not demonstrated. These findings complement previous studies of ENO2 regulatory mechanisms by providing an integrated candidate-prioritization and functional-validation framework. ENO2 remains a candidate metabolic biomarker and therapeutic target requiring validation in larger clinical cohorts and clinically relevant models.

## Supporting information

S1 File All raw images. (PDF)
