# Supplementary material for: Mechanistic insights into ENO2-driven glycolysis and tumor progression in hepatocellular carcinoma
Source: PLoS One. 2026 Oct 27;21(10):e0361057. doi: 10.1371/journal.pone.0361057 (PMC13712540; doi:10.1371/journal.pone.0361057)

Fig 7

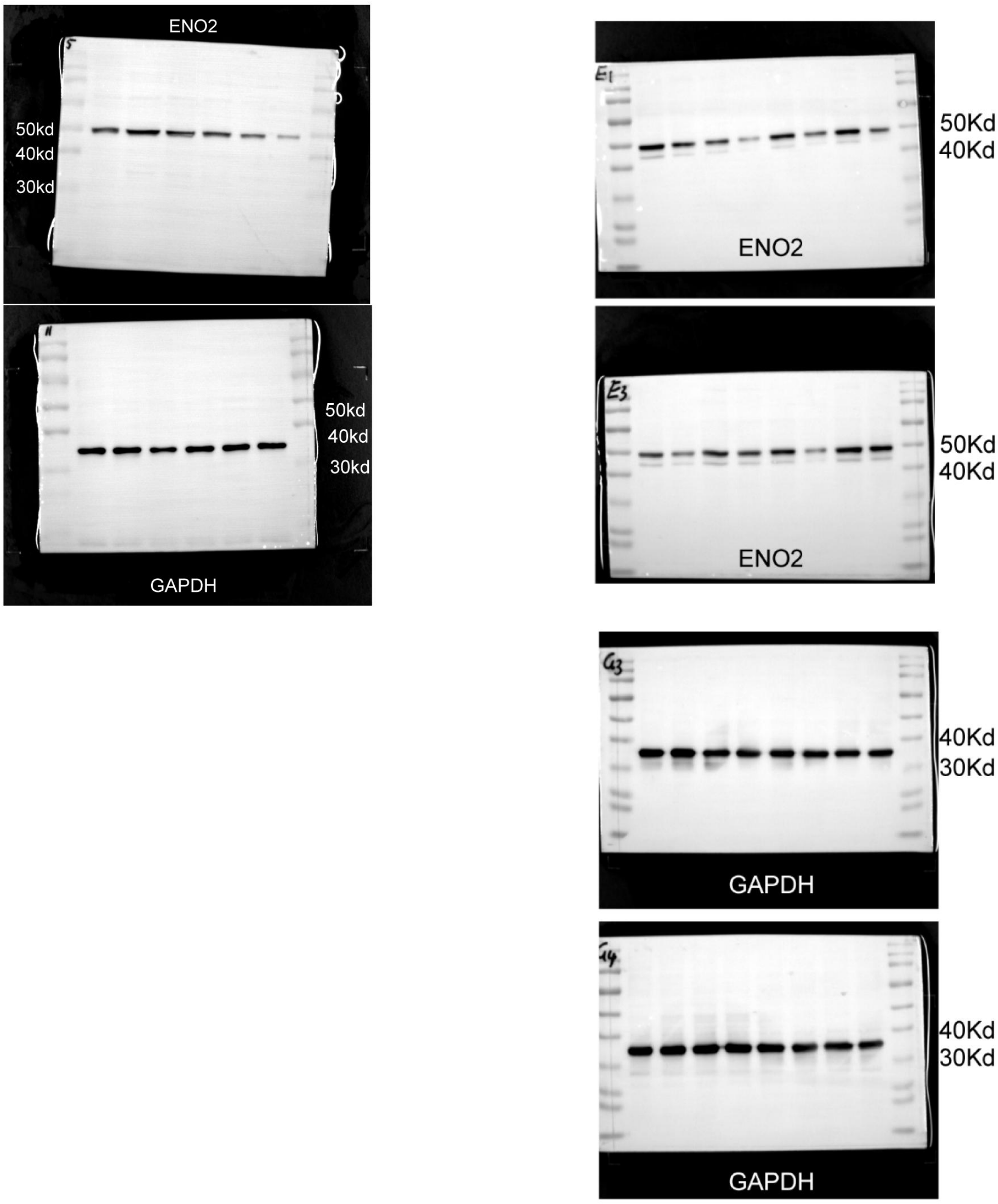
ENO2
50kd
40kd
30kd
50kd
40kd
30kd
GAPDH
50Kd
40Kd
ENO2
50Kd
40Kd
ENO2
40Kd
30Kd
GAPDH
40Kd
30Kd
GAPDH

Fig 8

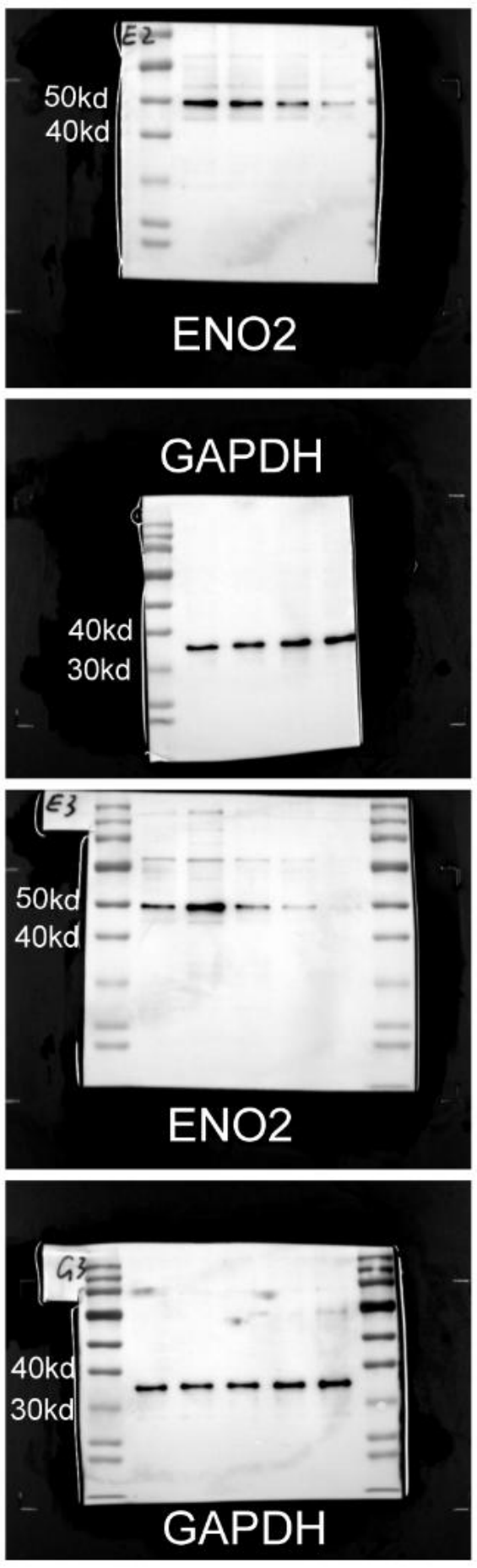

Fig 12

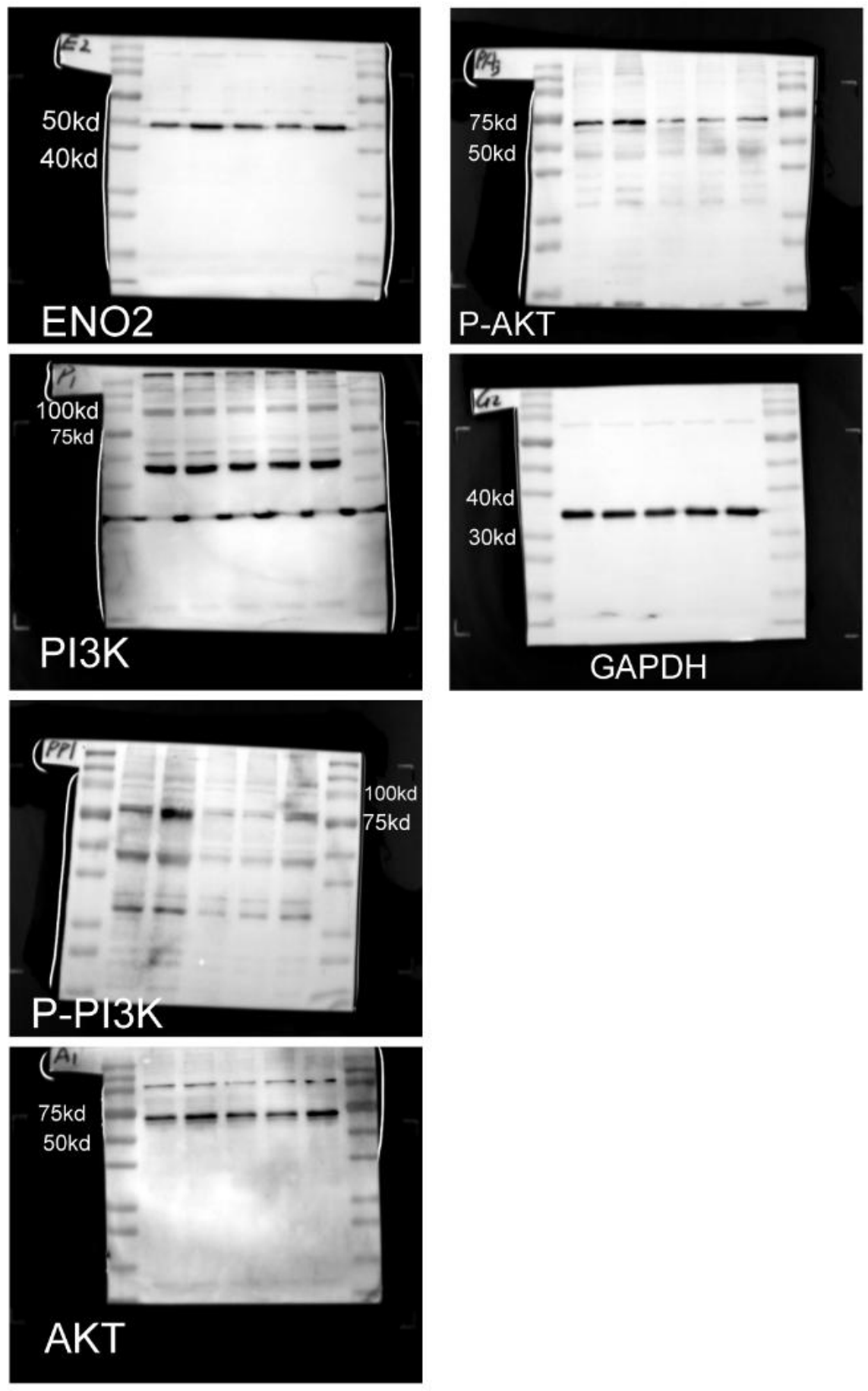

Fig11

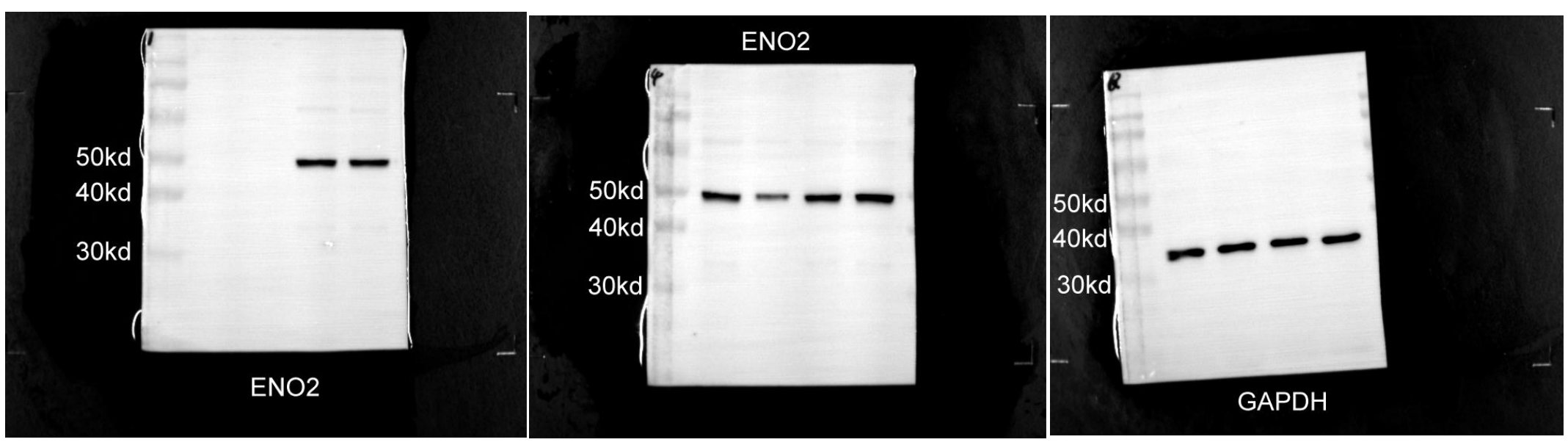

Supplement: Supplementary file 1 [file pone.0361057.s001.pdf]
